# Gold- or Silver-Nanoparticle SERS Platforms for Plasma-Based Diagnostics and AI-Driven Analysis

**DOI:** 10.3390/s26134131

**Published:** 2026-06-30

**Authors:** Gideon L. Elizur, Alexandre Canhoto, Gabriela Soares, Lucio Studer Ferreira, Eulália Pereira, Ricardo Franco

**Affiliations:** 1 Associate Laboratory i4HB—Institute for Health and Bioeconomy, Faculdade de Ciências e Tecnologia, Universidade NOVA de Lisboa, 2819-516 Caparica, Portugal; g.elizur@campus.fct.unl.pt (G.L.E.); am.canhoto@campus.fct.unl.pt (A.C.); 2 UCIBIO—Applied Molecular Biosciences Unit, Departamento de Química, Faculdade de Ciências e Tecnologia, Universidade NOVA de Lisboa, 2819-516 Caparica, Portugal; 3 ISCTE—University Institute of Lisbon, 1649-026 Lisbon, Portugal; gabriela.soares@iscte-iul.pt; 4 COPELABS, Lusófona University, Campo Grande 376, 1749-024 Lisbon, Portugal; 5 INESC INOV-Lab, 1000-029 Lisbon, Portugal; 6 LAQV/REQUIMTE—Laboratório Associado para a Química Verde/Rede de Química e Tecnologia, Departamento de Química e Bioquímica, Faculdade de Ciências, Universidade do Porto, 4169-007 Porto, Portugal; eulalia.pereira@fc.up.pt

**Keywords:** surface-enhanced Raman spectroscopy (SERS), plasmonic nanoparticles, gold nanoparticles, silver nanoparticles, plasma diagnostics, liquid biopsy, machine learning, artificial intelligence

## Abstract

Surface-enhanced Raman spectroscopy (SERS) has emerged as a highly promising analytical technique for disease diagnostics due to its exceptional sensitivity, molecular specificity, and ability to detect a broad range of biomarkers in complex biological matrices. This review provides a comprehensive overview of gold- and silver-nanoparticle-based SERS platforms for plasma disease diagnostics, covering advances in plasmonic nanostructures, biological sample analysis, biomarker detection, and AI-driven spectral data processing. Particular emphasis is placed on the application of SERS to clinically relevant biofluids, especially plasma, where the technique has demonstrated considerable potential for detecting diseases such as cancer, inflammatory disorders, and neurological conditions. The review also critically examines the major challenges currently limiting the clinical translation of SERS technologies. These include variability associated with substrate fabrication, matrix-induced signal fluctuations, limited interlaboratory reproducibility, and the lack of standardized protocols for spectral preprocessing and data analysis. Strategies proposed to address these issues are discussed, including comprehensive post-synthesis substrate characterization, optimization of biological sample preparation, advanced spectral preprocessing workflows, and the integration of machine learning and artificial intelligence algorithms to improve diagnostic robustness and reproducibility. Collectively, the advances summarized in this review indicate that SERS-based diagnostic technologies are rapidly progressing beyond proof-of-concept studies toward clinically applicable systems. Continued interdisciplinary collaboration and standardization efforts will be essential to bridge the remaining gap between experimental SERS methodologies and routine clinical implementation.

## 1. Introduction

Clinical laboratory diagnostics is a key pillar of the global healthcare management system. To be both efficient and effective, new techniques, equipment and devices that complement, improve, or substitute available laboratory diagnostic systems are necessary. Speed, sensitivity, selectivity, scalability, minimally invasive sampling, and point-of-care (POC) capability are among the desirable characteristics for any product developed and intended for modern diagnostics [1]. Contemporary diagnostic gold standards based on spectroscopy, chromatography, immunochemistry, and molecular biology are both effective and efficient; however, they do not meet all the criteria of ideal medical diagnostic systems [2].

Surface-enhanced Raman spectroscopy (SERS) is an established vibrational spectroscopic technique based on the inelastic scattering of monochromatic light by matter. It has potential utility in developing methods for highly sensitive and selective detection that can be adapted for rapid, accurate and less invasive clinical diagnostics [3,4]. SERS, via the plasmonic metallic nanoparticles (NPs) present, amplifies weak Raman signals by several orders of magnitude, enabling molecular fingerprinting and molecule detection of target analytes present in very low concentration, even in complex matrices such as biofluids [5,6,7].

Unlike tests based on variants of enzyme-linked immunosorbent assay (ELISA), polymerase chain reaction (PCR), mass spectrometry and many other conventional techniques, SERS requires minimal sample preparation, a less controlled environment, and a comparatively lower sample volume while offering a superior multiplexing capability and analytical speed [8,9]. SERS has been applied in biomedical research on neoplasms [10,11,12]; infectious diseases [13,14,15,16]; neurodegenerative and cerebrovascular disorders [17,18,19]; drug monitoring [20,21,22]; and toxicological assessments [23,24] in biofluids and other biological matrices, with many studies reporting the multiplexing potential of SERS [25,26,27,28]. 

Blood plasma is an important repository of valuable information about biological processes of clinical importance. It contains proteins, nucleic acids, lipids, metabolites, and associated biomarker components that reflect the extracellular environment of major organ systems relevant for clinical diagnostic and therapeutic purposes [5,29]. The complexity of plasma, however, compounds many analytical techniques, necessitating extensive sample preparation steps. SERS stands out as a versatile and viable technique for probing plasma to extract relevant clinical information because its substrates can be adapted for all types of biomarker evaluation, a distinction from PCR, which is limited to nucleotides, or ELISA, which is more specific to protein-based biomarkers. SERS-based immunoassays formatted according to ELISA or lateral flow assays (LFA) methods show comparative sensitivity, as has also been observed with PCR formats [30,31,32,33,34].

**Figure 1:**
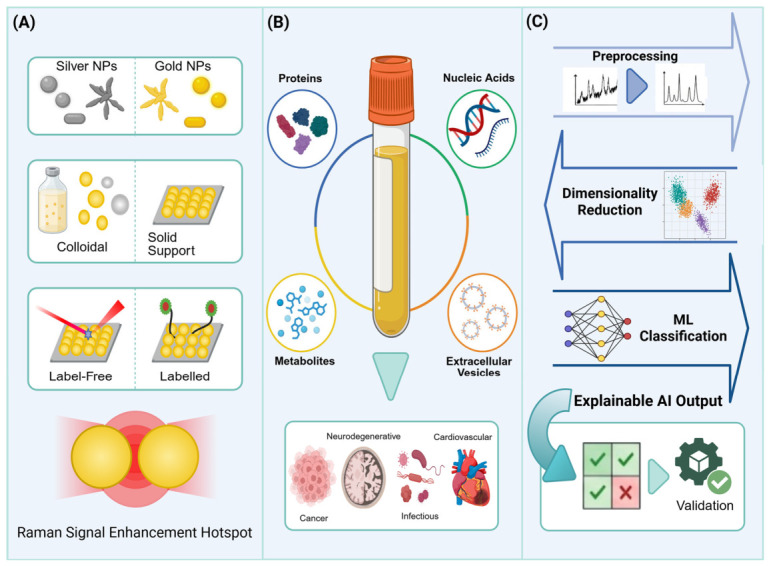
Schematic overview of the scope and main findings of the literature reviewed. (**A**) SERS fundamentals, enhancement mechanisms, and nanoparticle platforms used for biosensing applications. (**B**) Biomolecules contributing to plasma-derived SERS signatures and their relevance to disease diagnosis. (**C**) AI-driven models and analytical workflows for the processing, classification, and interpretation of SERS spectral data.

Despite the volume of available literature detailing SERS diagnostics, which includes several comprehensive reviews of SERS methodology [6,7,35,36], no existing review focuses specifically on plasma as the biological matrix, gold (Au) and silver (Ag) nanoparticles (NPs) as the plasmonic substrates, and chemometric methods encompassing the diverse traditional statistics and associated artificial intelligence (AI) algorithms for SERS spectra interpretation. This combination represents different strategies researchers are exploring to address SERS assay challenges arising from substrates, the biological matrix, and data interpretation platforms. The scope of this review is therefore defined by three parameters, namely plasma as the biological matrix, the nanoparticle substrate (AuNP and AgNP, including hybrid architectures), and the SERS data interpretation framework (including linear chemometrics, machine learning (ML) and explainable AI). Figure 1 presents a schematic overview of the scope and main findings of the literature reviewed.

The sections of this review are organised to provide a brief introduction to the principles and mechanisms of SERS enhancement and the direct/indirect detection strategies in Section 2. An introduction to AuNP and AgNP as SERS-active materials and their properties is highlighted in Section 3. Section 4 briefly highlights biomarkers in biological matrices, with a focus on blood plasma and matrix-derived challenges such as protein corona formation, the coffee-ring effect, and SERS result variability due to random aggregation when using colloidal suspensions. Section 5 provides an overview of clinically relevant SERS research using plasma as the biological matrix. It is organised by a detection modality that progresses from label-free single-mode strategies to labelled multiplexed immunoassays and multimodal biosensing systems across a range of oncologic and non-oncologic target analytes. A detailed presentation of the SERS data analysis pipeline—from spectral preprocessing through dimensionality reduction to machine learning classification and explainable AI methods—is covered in Section 6. Section 7 briefly discusses the challenges limiting the translation of SERS from the research laboratory to clinical utility. It also highlights opportunities that could determine the trajectory of the SERS research field. Conclusions are presented in Section 8.

## 2. Surface-Enhanced Raman Spectroscopy (SERS)

SERS is reputed to exhibit extreme signal enhancements responsible for the high sensitivity and specificity of vibrational fingerprinting. This section highlights the underpinning mechanisms of SERS enhancement and measurement approaches using either a label-free SERS or a labelled/tagged SERS format for target analyte detection.

### 2.1. Principles of SERS

When light is scattered due to electron cloud distortion upon interacting with the molecules of a material, intense elastic scattering (Raleigh scattering) mostly occurs without any associated energy transfer. A rare and less intense inelastic scattering (Raman effect) also results in an associated photon energy gain (anti-Stokes) or loss (Stokes) [6,37]. This difference in scattered photon energy, termed the Raman shift, provides a fingerprint unique to the respective molecules interacting with the incident photon, enabling accurate chemical identification. This interplay of scattered light, molecular vibration and energy transfer is the basis of Raman spectroscopy, which is an established vibrational spectroscopic technique with excellent molecular specificity for the determination of molecular conformation, adsorption orientation, and surface bonding in materials [29]. However, the low intensity of Raman signals due to the low probability of Raman scattering events (1 in 10^6^–10^9^ photons in comparison with Raleigh scattering), alongside fluorescent interference, hinders the optimal application of Raman spectroscopy in analyses involving complex matrices, as is common with clinical laboratory diagnostics [37,38]. The discovery of surface-enhanced Raman spectroscopy (SERS) based on Raman signal amplification of about 10^6^–10^14^ greatly extended the sensitivity and applicability of Raman spectroscopy [39,40].

**Figure 2:**
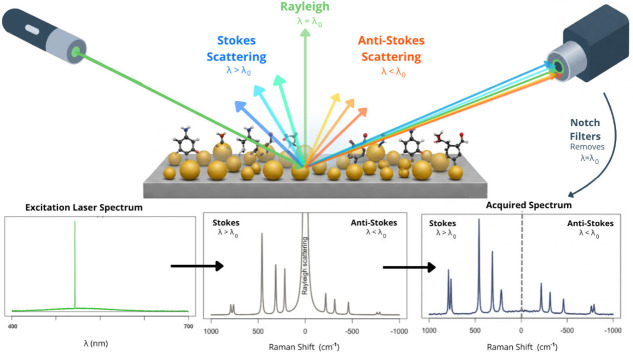
Illustration of SERS measurements based on Stokes/anti-Stokes scattering.

**Figure 3:**
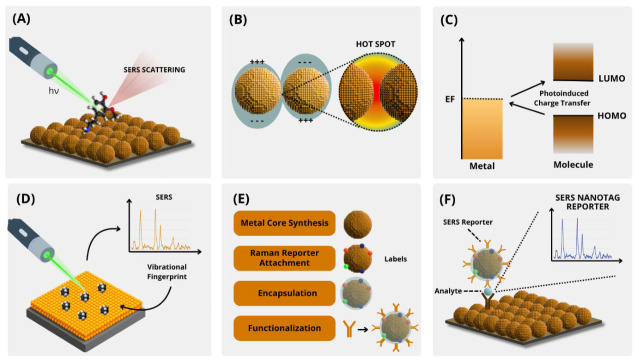
Schematic of SERS mechanisms. (**A**) Incident light interacts with molecules adsorbed on the surface of plasmonic nanoparticles. (**B**) Depiction of hotspots from the interparticle gap. (**C**) Charge transfer (CT) enhancement mechanism based on the energy difference between molecular orbitals. (**D**) Overview of label-free SERS measurement. (**E**) Labelled SERS substrate. (**F**) Utilising nanotags/probes in SERS measurements.

SERS achieves this high signal amplification by means of nanoparticle (NP) substrates derived from silver, gold, copper and other plasmonic metals as depicted in Figure 2. The delocalised conduction electrons at these NP surfaces undergo coherent oscillation in response to incident photons at specific resonance frequencies, a phenomenon termed localised surface plasmon resonance (LSPR). A dominant LSPR-dependent electromagnetic enhancement mechanism (EM) and a simultaneously occurring charge transfer or chemical enhancement mechanism (CT) are the widely accepted mechanisms contributing to the observed Raman signal enhancement in SERS [41]. In EM, the more prominent of the two mechanisms, LSPR, is key; it is influenced by the nanoparticle’s geometry, dielectric function, and the permittivity of the surrounding medium [37]. Under photon excitation, when the oscillating frequency of the NP electron cloud is in resonance with the frequency of the incident photons, a significant increase in the near-field amplitude at the NP surface occurs. Consequently, the incident electromagnetic field is enhanced by a resonance-driven dipole field emitted by the NPs. The closer the LSPR frequency is to that of the incident photon, the greater the field enhancement. In effect, analyte molecules either adsorbed on the NP surface or located very near (≤10 nm) the NP surface become intensely polarised by the excited surface plasmon, resulting in EM Raman signal enhancement reaching 10^8^–10^11^ [6,37,42]. Additionally, EM enhancement is further intensified at the points of contact and at the interparticle gaps between NP pairs and clusters in a distance-dependent manner (<10 nm), forming active sites or hotspots [9]. Because the LSPR frequency, linewidth, and near-field spatial distribution are a function of nanoparticle geometry, nanoparticles with anisotropic geometries contain a higher hotspot density than regular geometries, with an increase in hotspot density achieved by manipulating NP aggregation, anisotropic geometries and the incident photon (Figure 3A) [9]. These hotspots can generate a strong electric field with resultant enhancement factors (EFs) approaching 10^14^, allowing for sensitivity leading up to single-molecule detection (Figure 3B) [6,9].

In contrast, the CT enhancement mechanism involves electron transfer between the analyte molecules and the plasmonic NPs due to chemical bonding and the formation of new electronic states. An increase in molecular polarizability occurs, depending on the alignment and interaction between the molecule’s highest occupied molecular orbital (HOMO)/energy level with electrons, its lowest unoccupied molecular orbital (LUMO)/lowest empty energy level, and the metal’s Fermi level (*E_F_*), as illustrated in Figure 2. Instead of plasmons, there is a two-way charge transfer between the analyte molecule and NPs, like anti-Stokes Raman scattering. A first-layer effect arises as a consequence of CT chemisorption dependence, resulting in SERS enhancement only at the first layer of molecules, which are directly in contact with the NP substrate. Notwithstanding, the enhancement from resonance scattering, the enhancement from plasmon resonance, increased molecular polarizability resulting from adsorption, and the charge transfer resonance of the metal and molecule complex all contribute co-dependently to the chemical enhancement, adding an extra 10^2^–10^3^ to the overall enhancement [37,41].

A simplified overview of the SERS mechanism is what has been described. Detailed explanations on SERS principles and enhancement mechanisms are available from many other sources in the literature [35,43,44].

### 2.2. Direct/Label-Free SERS

To exploit the excellent signal enhancement of SERS, two alternative approaches can be employed for qualitative and quantitative SERS detection of biomarkers in plasma and other biological matrices. The indirect or labelled/tagged SERS approach (Figure 3E,F) measures the specific signals of chromogenic compounds called Raman reporters attached to the plasmonic metal, while the direct or label-free SERS approach identifies intrinsic spectral bands generated by the analyte molecules in direct response to the laser-excited NP field effect (Figure 3C) [5,6,45]. When no bioreceptor is involved as a capture agent in the direct SERS format, the different functional groups of molecules present in the analyte sample contribute collectively to the resulting SERS spectra, providing a complex molecular signature specific to the sample, from which an identification/classification is deduced for a target biomarker or a particular pathologic condition. The label-free approach offers several practical advantages that make it particularly suited to clinical plasma analysis. It is the simplest and fastest configuration of SERS approaches, a quality that directly supports point-of-care (POC) applications. Direct SERS also require no NP derivatisation with reporter molecules, which can reduce assay complexity and preparation time. Additionally, it can be performed with or without a specific biomarker target. The different analytes, ranging from small molecules to cells and tissue samples, interact with the plasmonic fields by direct adsorption onto bare NP surfaces [45] or through capture by a bioreceptor immobilised on the NP. Regardless of whether a bioreceptor is incorporated, label-free SERS for disease diagnostics relies critically on traditional chemometric and advanced algorithms (including AI, ML, deep learning, DL) analysis of the resulting spectra.

Regardless of whether a bioreceptor is incorporated, label-free SERS for disease diagnostics relies critically on chemometric and machine learning analysis of the resulting spectra. Instead of identifying a single peak intensity, the label-free approach extracts diagnostic information from differences in the overall spectral profile between healthy and disease cohorts, especially when large datasets are involved [42], as illustrated in Figure 3. Alongside these complex computational approaches, small datasets can be manually interpreted from simple calibration models [6].

**Figure 4:**
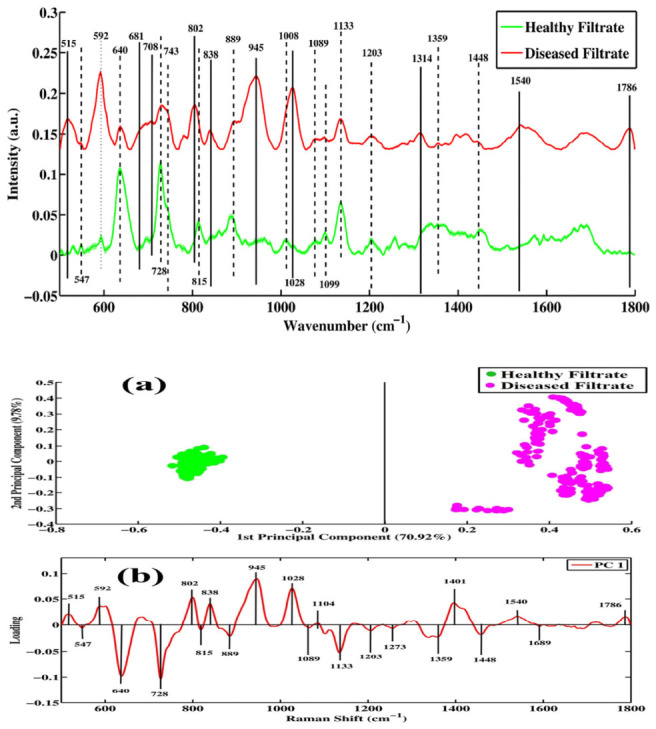
(**Top**) Mean SERS spectra of filtrate portions of healthy and tuberculosis-positive blood serum samples with standard deviation. (**Bottom**) Pair-wise PCA analysis. (**a**) Scatter plot and (**b**) loadings between SERS spectral datasets of filtrate portions of healthy and tuberculosis disease samples. [Source: Reprinted from Kamran Ali et al., 2024 [15]. 2024 American Chemical Society. Creative Commons Attribution Non-Commercial 3.0 Unported (CC-BY-NC)].

A typical example of SERS measurements and spectra in the label-free approach is depicted in Figure 4 (Top), where chemometrics compares SERS spectra datasets from physiologic and pathologic samples to predict health conditions using significant bands for calibration and classification (see Figure 4 (Bottom), (a) and (b)). After simple pretreatment (filtration) of serum with a 50 kDA filter, a classification accuracy of 97% with 97% specificity, 98% sensitivity, and an area under the receiver operating characteristic curve (AUC) of 0.74 was achieved by principal component analyses (PCA) combined with partial least squares discriminant analysis (PLS-DA) for discriminating healthy serum samples from pathologic tuberculosis serum samples using unlabelled spherical AgNP colloids [15]. In another example, Gao Siqi and team utilised AgNP colloids in a simple SERS assay to detect liver and prostate cancers in serum, focusing on the coffee ring formed [46]. The successful discrimination between healthy and typhoid-fever-infected serum samples was also achieved using colloidal AuNPs in a label-free SERS assay by Ditta and colleagues [47]. Using aggregation agents to increase hotspot density in spherical AuNP suspensions, Turzhitsky et al. detected relevant opioids in urine at picogram concentration [48]. Diverging from colloidal suspensions as SERS substrates, Chen Sheng et al. deposited colloidal AgNPs (nanospheres) on a cellulose triacetate porous membrane to form a 3D-structured SERS substrate that enabled the label-free detection of kidney cancer in blood plasma [49]. Similarly, by fabricating a SERS substrate with silicon nanowires and AgNPs (SiNW@Ag) to increase hotspot density, an EF of 4.0 × 10^8^ was achieved that enabled the label-free detection of dopamine in blood plasma samples of depressive patients, with a 4.37 × 10^−12^ M limit of detection (LOD) [50].

Conversely, rather than using spherical NPs, increased hotspot density from anisotropic geometries was explored to fabricate a SERS substrate from star-shaped AgNPs (nanostars) on a filter paper support matrix. This was utilised to assess interspecies and intraspecies discrimination of *Acinetobacter baumannii* and *Klebsiella pneumoniae* strains, where principal component analysis (PCA) and partial least-squares discriminant analysis (PLSDA) allowed interspecies differentiation with specificity in the range of 0.93–1.0 and 0.75–1.0 sensitivity. Intraspecies classification for *K. pneumoniae* strains was in the range of 0.73–1.0 [14]. While the simplicity of the label-free SERS approach is challenged by interference from high molecular weight constituents in biological matrices, impacting its sensitivity and selectivity, it remains a preferred method for analysing samples that have no existing biomarker or whose biomarkers are non-specific, making it relevant for biopsies [46,51,52] and other methods of clinical detection, such as drug monitoring, organ injury, and neurodegenerative disorders [20,53,54].

### 2.3. Indirect/Tagged SERS

Some chromogenic compounds called Raman reporters produce SERS signals that are distinct, intense, and stable when adsorbed on AgNP/AuNP and other NPs. During indirect SERS, the signal is extrinsic to the target analyte, i.e., rather than measuring the signals from the sample molecules, it is the perturbed Raman signals from the chromophores such as malachite green isothiocyanate (MGITC), 4-mercaptobenzoic acid (4-MBA), 5,5′-dithiobis(2-nitrobenzoic acid) (DTNB), and others present that get detected and analysed to provide information about the sample of interest [3,5]. The coupling of these Raman reporters, which are mostly organic dyes, to the NP surface with additional targeting ligand functionalisation creates labelled/tagged SERS substrates (if on a solid support) or SERS nanotags/nanoprobes (colloids) with high selectivity and sensitivity, superior to the direct approach. Generally, NPs form the core of the nanotags responsible for amplifying signals; reporter molecules ease spectral identification due to their distinct signatures; a protective polymer/inorganic/bio-molecule shell coating improves stability and biocompatibility; and a biorecognition element enhances specificity [36], which is illustrated schematically in Figure 3E,F. The advantage of SERS nanotags over existing fluorescence/colorimetric labels is that they have high photostability over a longer measurement time, a single laser wavelength excitation for different SERS nanotags, and reduced autofluorescence in biological matrices [6]. Also, the Raman scattering cross-section of individual reporter molecules and the number adsorbed per NP determines the brightness of the SERS nanotags [6]. In disease diagnostic applications, the non-overlapping narrow bandwidth of the different reporters means highly customisable SERS nanotags can be designed for single or multiplex detection of a variety of biomarkers. This is significant for SERS optimisation processes aimed at addressing some of the identified challenges pertaining to poor reproducibility, the biological matrix effect, and other issues affecting SERS-based diagnostics [55,56].

A very important analytical tool for biochemical studies and clinical diagnosis is the immunoassay with different formats [55,57]. In singleplex or multiplexing, supported by a narrow full width at half-maximum (FWHM) of vibrational Raman bands of easily customisable substrates, sandwich immunoassay formats can be followed similar to ELISA, with the difference being that the capture probe is an antibody (Ab) coated on an NP surface or a solid substrate, and the label probe consist of AuNP/AgNP conjugated to a Raman reporter and an antigen-specific layer of antibodies [56]. A demonstration of this labelled SERS immunoassay detected neutrophil gelatinase-associated lipocalin (NGAL) and cystatin C (Cys C), two acute kidney injury biomarkers, at a nanogram concentration using a custom DNA-Aptamer AuNP probe [58]. In combining 4-mercaptobenzonitrile (4MBN) and 4-mercaptophenylboronic acid (4-MPBA) as Raman reporters in a SERS tag and SERS substrate sandwich format, Lu Dechan and team fabricated a labelled SERS platform with high sensitivity and specificity for carcinoembryonic antigen (CEA) [12]. A device utilising custom nanotags from different Raman reporters and antibodies was developed for high-throughput multiplex detection of septicaemia-causing pathogens in large clinical sample volumes, achieving detection limits of single colony-forming unit (CFU) [59].

**Figure 5:**
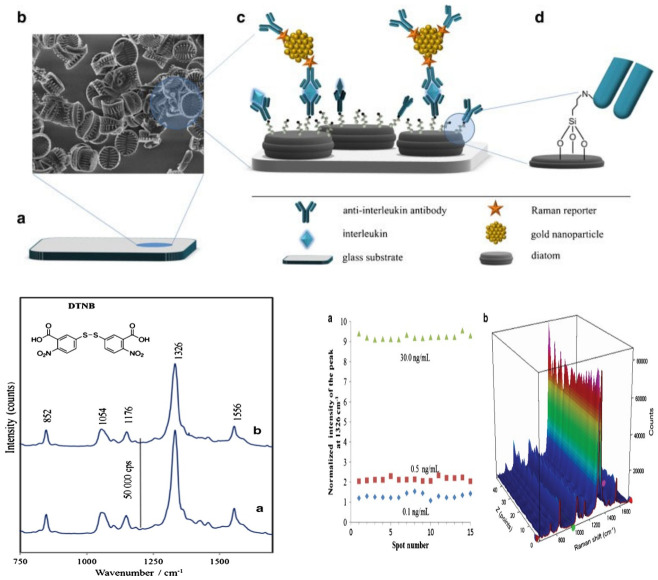
(**Top**) Schematic illustration of the SERS-based immunoassay: (**a**) substrate design and fabrication; (**b**) SEM characterisation; (**c**) bioconjugation of antibody; (**d**) close-up illustrating the bonding to the antibody. (**Bottom Left**) (a) SERS spectrum of DTNB@AuNPs. (b) SERS spectrum of DTNB@AuNPs@Ab. (**Bottom Right**) (**a**) Representative graph detailing results reproducibility. (**b**) Representative two-dimensional SERS spectra from spatial spots coverage [Source: Reprinted from Kamińska et al., 2017 [60]. 2017 Springer Nature. Creative Commons Attribution 4.0 (CC-BY-4.0)].

As shown in Figure 5a–d, a detection probe (AuNPs@DTNB@anti-IL8) was fabricated from AuNP colloids, the 5,5′-dithiobis(2nitrobenzoic acid) (DTNB) Raman reporter, and anti-interleukin 8 (anti-IL8). Together with a capture probe of immobilised anti-IL8 antibodies on amino-modified diatom frustules substrate, a SERS sandwich immunoassay was used to detect the inflammatory cytokine interleukin 8 in blood plasma, utilising two different substrate support matrices. Sequential SERS measurements at key steps of the substrate fabrication and the immunoassay phases for data acquisition, quality control and result validation, enabled proper data interpretation using chemometrics (Figure 5, Bottom). When comparing the detection efficiency of substrate supports made from diatom biosilica with those made from glass, LODs of 6.2 pg.mL^−1^ and 2.5 ng.mL^−1^, respectively, were achieved [60].

SERS-based immunoassays have become widespread [6,56], allowing for novel applications such as multimode sensing where the specificity and enzymatic activity common in immunochemical detection can be combined with the advantages of SERS-based lateral flow assays (SERS-LFAs) [61]. This multimode SERS strategy was demonstrated by Liang Penghui et al., who adopted a colourimetry-SERS configuration to detect SARS-CoV-2 antigens at picomolar levels using an LFA containing antibody-functionalised 4-MBA on an Ag core and Au shell [62]. From the foregoing, the choice to follow either the labelled or unlabelled SERS approach for diagnostic purposes is influenced by the sample type and the characteristics of the target analyte, with some experimental parameters better suited for either strategy [38]. A comparative overview detailing key features of direct and indirect SERS assays in plasma is highlighted in Table 1.

## 3. SERS-Active Nanoparticles

Following the brief introduction to SERS principles and enhancement mechanisms, this section introduces AuNPs and AgNPs as the predominant SERS-active nanomaterials alongside hybrid nanostructures that combine the complementary attributes of Au and Ag with each other or with other metals. Additionally, NP geometries and fabrication strategies are highlighted, together with the properties that enable SERS analytical performance in plasma-based diagnostics.

### 3.1. Properties of SERS-Active Nanoparticles

The most critical components in SERS are the plasmonic metals with ideal properties integral to the associated signal enhancement [63]. These properties include tuneable LSPR, high electrical conductivity, fluorescence quenching or enhancement, and light scattering capability [64]. Gold (AuNP) and silver (AgNP) nanoparticles have suitable plasmonic properties and hence are the most common materials used for bioanalytical SERS [7,9,63]. Notably, they can be excited at wavelengths ranging from the near-visible to the near-infrared region, which is significant for both in vivo and in vitro studies [9,37,38].

In terms of morphology, and this cuts across AuNPs, AgNPs and hybrid nanostructures, the stable structure, simple synthesis, and ease of modification result in spherical AuNPs and AgNPs forming the core of most SERS substrates/tags [65]. However, anisometric nanoparticles possess intrinsic properties that make their use advantageous in many SERS applications. Table 2 summarises the physicochemical and analytical properties of AuNP, AgNP, and hybrid nanoparticles. Nanorods exhibit a strong longitudinal plasmonic band at NIR and a weak transverse band near the visible region, with length-to-width tunability, making them extensively investigated one-dimensional anisotropic NPs [65,66]. The tip-selective growth of nanorods can be used as a template for the synthesis of nanowires, another class of anisotropic shapes. Another shape, nanobipyramids, due to having multiple sharp points, display stronger EF and SERS signals than nanorods, making them suitable for imaging and other diagnostic purposes. Nanocages possess porous walls with a hollow interior, suitable for colourimetry. Nanoplates with a triangular shape are characterised by sharp corners, large surface area, and high electromagnetic field enhancement. They also exhibit broad electrical and optical properties, with plasmon bands in the visible and NIR region, with readily functionalised surfaces, especially from molecules with thiol functional groups. Their high-yield synthesis and ease of functionalisation make them suitable for in vivo imaging and other diagnostic applications [65,66]. What is obvious is that there are myriad regular and novel NP geometries, each exhibiting a range of properties suitable for specific assays.

The diverse applications of noble metal NPs stem from unique optical, magnetic, catalytic, and therapeutic properties that emerge under nanoscale spatial confinement, where electronic structures transition from semicontinuous to discrete. Specifically, gold nanoparticles with a core diameter of 3 nm or larger adopt a face-centred-cubic (FCC) structural framework with a semicontinuous electronic profile, enabling coherent collective electronic oscillations known as surface plasmon resonance [67]. Conversely, when dimensions fall below the 3 nm threshold, the particles cannot support plasmon resonance; their resulting discrete electronic states trigger molecular-like properties, classifying them as nanoclusters. These distinct electronic configurations, combined with exceptionally high surface-to-volume ratios, render the physicochemical properties of noble metal nanoparticles highly sensitive to absolute size, elemental composition, intraparticle atomic packing, and interparticle hierarchical arrangement. Consequently, this profound sensitivity has driven the development of advanced, precise synthetic methodologies to control these property-dictating attributes [67].

The SERS-optimised dimensions across nanostructures follow a clear size–function relationship [67,68]. Nanospheres perform best with core diameters between 30–80 nm, a range that preserves strong dipolar plasmon modes while avoiding both quantum damping at small sizes and multipolar scattering at larger ones. Nanorods require more than a one-dimensional constraint: their 20–40 nm core diameters maintain a clean longitudinal dipole resonance, their 50–150 nm lengths tune the LSPR to match the excitation wavelength, and their <5–15 nm tip radii create intense localised hotspots. Nanoprisms rely on thin 10–30 nm cores to support strong in-plane dipole modes, 50–150 nm edge lengths to set the resonance wavelength, and <5–10 nm tip radii to maximise field enhancement at their vertices. Finally, nanostars, whose performance is dominated by curvature, achieve their strongest hotspots when their tips are sharpened to <2–10 nm, enabling extreme electromagnetic localisation [67,68]. What is obvious is that NP size determines its light-absorption and scattering cross-sections and the efficiency of LSPR. Geometric isotropy or anisotropy determines the spatial distribution of plasmonic energy, with anisotropic shapes concentrating the electromagnetic field into highly intense “hot spots” at sharp vertices rather than distributing it uniformly like isotropic spheres.

### 3.2. Gold Nanoparticles

In addition to the plasmonic properties already mentioned, gold nanoparticles (AuNPs) are anti-photobleaching and non-toxic, with relative chemical stability in complex matrices, which further promotes their use in biomedical applications [64,69]. While AuNPs are noted for lower SERS enhancement intensity compared to AgNPs, they are considered ideal for diverse biological sensing platforms given the associated enhancement in the red spectral region [9,37]. Notably, spherical and anisotropic AuNPs are readily synthesised via several top–down/bottom–up techniques, enabling the tuning of LSPR to desired wavelengths [66,70]. In particular, anisotropic geometries such as Au nanostars, which have multiple thin branches on a small core, produce not only plasmonic absorption in the near infrared (NIR) range, favourable for tissue optical applications, but also surface plasmons several orders of magnitude stronger than their spherical counterparts [71]. Additionally, AuNPs possess surfaces that are readily functionalised with diverse chemical moieties, which makes them suitable for labelled SERS assays [64,72]. All these properties give insights into why AuNPs are a recurring component of many SERS-based sensors. In terms of size, a study examined the impact of NP size on SERS signal intensity for bioanalysis by comparing spherical AuNPs of 150 nm and 40 nm diameters in a label-free SERS assay using MCF-7 breast cancer cells. The result favoured the use of larger NPs for optimal SERS signal in the NIR and IR regions [72].

### 3.3. Silver Nanoparticles

Silver nanoparticles (AgNPs) are the second of the two most featured plasmonic substrates used in diverse SERS analyses. They exhibit all the associated properties of optimal SERS substrates in addition to possessing a significantly higher extinction coefficient and intrinsic SERS EF than AuNPs [35]. This high EF at excitation wavelengths ranging from the 400 nm region and above promotes its use in numerous biosensing assays [9]. The fact that AgNPs are less stable than AuNPs, prone to oxidation, and cytotoxic does not limit their usage in SERS and other analytical techniques. Rather, optimisation and synthesis strategies have mitigated these effects, maintaining their continuous use for different applications [14,73,74,75,76].

### 3.4. Hybrid Nanostructures

The need to enhance the advantages of either or both gold/silver NPs by combination with each other, or with other metallic nanomaterials, informs the fabrication of hybrid nanostructures. In hybrid nanostructures derived from only Au and Ag, the core is most often Au, providing chemical stability and a scaffold for thiol-based bioconjugation, with an Ag shell to maximise SERS enhancement [5,36,77]. These architectures are engineered to leverage the complementary attributes of disparate materials, notably the chemical resilience of gold (Au) alongside the superior enhancement capabilities of silver (Ag). Prevalent hybrid configurations include core–shell architectures made with Au and Ag only combined as either Au@Ag, or Ag@Au, benefiting from the associated Au stability and the high EF of Ag. Other heterogenous and multicomponent configurations combine either one of Au and Ag, or both with other select metals or compounds. Studies find that hybrid core–shell nanostructures exhibit strong LSPR, with the core radius and shell thickness determining the SERS enhancement [66]. Hence, hybrid nanostructures are relevant in plasma-based diagnostics, where the importance of stability, reproducibility, and high sensitivity is emphasised. Hybrid nanostructures through custom engineering can regulate the adsorption of biomolecules, mitigate non-specific interactions, and enhance the consistency of spectral data across diverse samples [5,36,77]. The fabrication of a hybrid Ag-Au bimetallic plasmonic substrate with 10^7^ SERS EF, subsequently labelled with select Raman reporter molecules, enabled the multiplex detection of three different interleukins at  pg·ml^−1^ levels in blood plasma using a microfluidic device [78].

A different class of hybrid nanomaterials are hierarchical nanostructures possessing a large reaction interface in a select surface area, allowing for superior biomolecular detection, catalyst charge transfer, metal ion release, and cavities effective for microbial capture. Their adjustable porosity, packing density, and controlled stability make them suitable for biosensing applications [79], offering advantages for SERS assays due to ultra-high sensitivity and low detection limits through pure EM amplification, as well as large area uniformity, excellent reproducibility, and practical reusability.

Recently developed hybrid architectures composed of noble metal Au/Ag NPs coupled with two-dimensional (2D) materials such as graphene, transition metal carbides/nitrides (MXenes), or transition metal dichalcogenides like molybdenum disulfide (MoS_2_) represent an important advancement in surface-enhanced Raman spectroscopy (SERS), yielding exceptional sensitivity through a synergistic dual enhancement mechanism [80,81]. The primary contribution arises from the classical electromagnetic mechanism (EM) localised at the noble metal domains. Upon laser excitation, the Au/Ag nanoparticles sustain localized surface plasmon resonance (LSPR), generating intensely amplified localised electromagnetic fields, or “hot spots”, at the nanostructured junctions that scale the Raman scattering cross-section by several orders of magnitude [82,83]. Concurrently, the integrated 2D material sub-layer provides a complementary chemical enhancement mechanism (CM). Owing to their unique electronic band structures and high surface area, materials like graphene, MXenes, or MoS_2_ engage in rigorous interfacial molecular interactions with adsorbed analytes. This proximity facilitates efficient photo-induced charge transfer pathways between the highest occupied/lowest unoccupied molecular orbitals (HOMO/LUMO) of the analyte and the Fermi levels of the 2D substrate. Electronic coupling alters the molecular polarizability of the target, effectively mitigating fluorescence background noise and preserving quantitative linearity while working in tandem with the metal-driven EM framework to drastically lower detection limits [82,83].

### 3.5. Anisotropic Nanoparticles Tag/Substrate

SERS substrates, whether in solid or colloidal form, are expected to possess an ideal large surface-to-volume ratio, high batch reproducibility, and most importantly, high EF, key determinants of the sensitivity and associated limit of detection (LOD) achievable by specific substrates [63,84]. As the physical properties of the NPs impact the degree of signal enhancement, only select NPs will facilitate a significant enhancement effect from the visible to the near-infrared (NIR) or infrared (IR) excitation wavelengths usual in SERS bioassays [72]. It then becomes necessary to optimise NP geometry and composition for maximum effect, therefore addressing the need for nanostructures with concentrated hotspot densities, in acknowledgement of single nanospheres generally being poor signal enhancers [9]. The feasibility of custom substrate fabrication for specific tasks is made possible by advances in synthesis and substrate fabrication methods, thereby expanding the utility of SERS-based assays [36]. 

As already stated, the synthesis route for all types of NPs follows the top–down or bottom–up approach, in line with mechanisms that may be physical, chemical or biological [66,76]. For high-yield spherical NPs, the bottom–up chemical reduction synthesis route is the most ubiquitous, where the Au or Ag salts are reduced in a one-pot synthesis to form monodispersed AuNP spheres [58,85]; AgNP spheres [86,87]; or spherical Au-Ag hybrids [12,88]. Non-spherical NPs, known to possess much higher EF than their spherical counterpart, can also be synthesised through a similar one-pot chemical reduction synthesis. However, for better control over NP morphology, the seed-mediated chemical synthesis route is preferred [66,71]. In the seed-mediated synthesis route, in contrast to fabricating NPs from kinetically controlled growth, a more controlled tuning of NP size and shape is achieved by using already synthesised NPs (seeding solution) of suitable size as templates in a reaction mixture (growth solution) that also contains reducing agents and capping ligands. Since it is energetically favourable for NPs to form on the seeds, a highly monodispersed final metal NP with a narrow size range is achieved [67,89]. The high tunability of the seed-mediated growth synthesis route has made the process suitable for fabricating single-shell NPS; Au@Ag NPs [90,91]; and multi-shell NPs [92,93].

**Figure 6:**
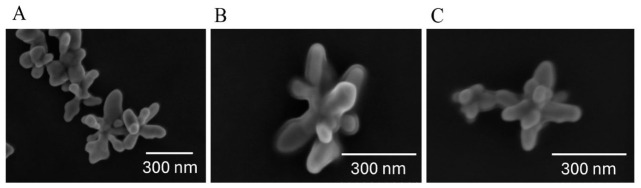
Scanning electron microscopy (SEM) representative micrographs illustrating the morphological characteristics of native AgNSs, or plasma–AgNS incubates. (**A**) AgNSs in their native form. (**B**) AgNS incubated with plasma for 15 min. (**C**) AgNS incubated with plasma overnight [Source: From Freitas et al., 2025 [17]. 2025 MDPI Creative Commons License (CC-BY)].

What stands out is that branched NPs that possess multiple branches, such as nanostars, are an important type of nanostructure, exhibiting high, tunable LSPR and SERS enhancement generated at the core and especially at the tips. Although they are not as monodispersed as other shapes, their high EF even in single-particle measurements makes them viable as a SERS substrate for disease diagnosis [65,66]. The EFs of many geometries are shown in Table 3. Nanospheres have a lower EF than the anisotoropic geometries shown in Table 3, and they most often require induced aggregation from an extrinsic agent, or from components in the reaction medium/matrix. To generate hotspots, individual nanostars are replete with sharp tips acting as hotspot nodes, and therefore they require no aggregation to generate intense enhancement fields. As an example, Freitas et al. utilised a one-pot chemical reduction process to synthesise AgNP nanostars with an average 99 nm arm length and 186 nm tip-to-tip length (Figure 6). By exploiting incubation time, the mitigation of a protein corona formation was achieved (seen as a low-contrast film in Figure 6B,C), noted for confounding SERS measurements. This enabled the use of a simple aluminium foil support for a label-free SERS assay for stroke detection [17].

Conversely, the seed-mediated synthesis of AuNP nanostars was used in fabricating a SERS immunoassay detection system with pg.mL^−1^ LOD. The tag, made from AuNP (nanostars) and Raman reporters (4-mercaptobenzoic acid, MBA, and 5,5-dithio-bis-(2-nitrobenzoic acid), DTNB) with antibodies (AuNP@MBA@anti-Ab and AuNP@DTNB@anti-Ab), used in combination with an antibody-functionalised 43.5 µm regenerated cellulose-based hydrogel (RCH) microfluidic capture platform, achieved differentiation between horseradish peroxidase (HRP) *Plasmodium falciparum* His-Rich Protein 2 (*Pf*HRP2). Through optimising the ligand coupling process, the reagent concentration, the molar ratio of AuNP and antibody, the reaction time, and the pH, a 5-fold increase in antibody biological activity of the nanostar SERS tag was achieved [57]. The citrate reduction and hydroxylamine reduction methods were used to synthesise respective AgNP nanospheres and nanostars, which were subsequently deposited on both a filter paper and a regular office paper designed with wax-printed wells to form a novel paper SERS substrate. Based on the SERS performance of paper substrate support (office paper vs. filter paper) and AgNP geometries (nanospheres vs. nanostars), office paper had superior uniformity per deposited AgNP density, requiring one-third the volume of AuNPs to detect the targeted analyte. An EF of 8.4 × 10^6^ (office paper) and 2.1 × 10^6^ (Whatman no. 1 paper) was obtained for spherical AgNPs, while the EF for AgNP nanostars was 3.0 × 10^7^ (office paper) with the same AgNP density. This further highlights the excellent enhancement from anisotropic NPS compared to spheres, in addition to showing how the choice of substrate support can impact SERS measurements [94].

Either route has also been utilised to synthesise several other anisotropic morphologies [95,96,97]. A specific example is the synthesis of AuNP (nanorods) via seed mediation, used for fabricating an AuNP@SiO_2_@MGITC@Ab tag, subsequently utilised to detect interleukin 5 (IL-5) in sputum [98]. Similarly, anisotropic AuNP (pyramids) encoded with three Raman reporters—4-ATP, 4-nitrothiophenol (NTP), and 4-methoxybenzyl mercaptan (MATT)—and DNA-framed aptamers for prostate-specific antigen (PSA), thrombin, and mucin-1 were synthesised to form a controllable multiplex SERS substrate; the aptamer-encoded AUNP tag was used for the simultaneous detection of the mentioned biomarkers in serum at attomolar LOD [99]. Additionally, physical methods such as photolithography and electron-beam evaporation also feature as fabricating routes for anisotropic NPs such as nanowires [18] and nanorods [100]. Table 3 lists common synthesis methods for different NPs.

## 4. Blood Plasma Matrix, Biomarkers, and Matrix Interference

SERS has emerged as a viable clinical diagnostic technique for probing biological matrices with high sensitivity, specificity and multiplexing capability comparable or superior to conventional laboratory methods. This section briefly introduces the different types of biological matrices that can be analysed with SERS, listed in Table 4. Also introduced are the constituent biomarkers within respective biological matrices targeted in SERS for disease detection.

### 4.1. Biological Matrices

Biological matrices are the source of biomarkers targeted in diagnostic SERS analyses, ranging from complex, protein-rich biofluids such as blood plasma and cerebrospinal fluid (CSF), through cellular and tissue specimens, to structurally heterogeneous solid matrices including bone, tooth enamel, and keratinous appendages such as hair and fingernails [5,101]. As repositories of vital anatomical, physiologic, and pathologic information, their applicability extends beyond disease diagnostics to include forensic and toxicological investigations for medical or legal purposes [5,102]. Solid specimens like bones, teeth, fingernails, and hair, widely known to endure environmental degradation, are mostly exploited in forensic SERS assays for crime investigations [102,103]. Health status from normal/abnormal levels of these sweat-based biomarkers can be inferred via regular SERS assays [104,105], or novel wearable SERS sensors [106,107]. Human tears have served as clinical samples for SERS-based detection of Alzheimer’s [108] and diabetes [109], among other diseases. Sputum and saliva have been samples in diagnostic SERS for pathogenic infections [110]; inflammatory agents [87]; periodontal diseases [111]; systemic autoimmune diseases [19,112,113]. SERS has been used to probe urine samples to evaluate kidney function through the variation in detected biomarker content levels [113,114,115]. Cerebrospinal fluid (CSF) has been used to detect pathogenic infection of the CNS [116,117]; neoplasms [118]; and other disorders of the CNS using a variety of AuNP/AgNP substrates. Given the complexity of biological matrices and the variation in biomarker quality and quantity, an understanding of biological matrix properties and composition informs their suitability as samples for different analytical procedures [119]. A summary of different biological matrices, their complexities, and their advantages and disadvantages is highlighted in Table 4.

Of all the biological matrices, blood-based samples (whole blood, plasma, serum) are the most used across all disease diagnostics [120]. Whole blood comprises blood cells (erythrocytes, leucocytes, and thrombocytes) suspended in plasma and contains biomarkers from almost all the body organs [40,120,121]). Serum is whole blood completely separated from blood cells. Clinical plasma is the liquid blood portion devoid of red and white blood cells, while retaining the coagulating factors, hence has higher protein content than serum [122,123]. While serum is less rich in proteins, hence having a reduced matrix effect on metabolite adsorption, plasma contains the full complement of blood proteins. There is no consensus on which is better as a clinical biological matrix [122]. Taken together, they contain the most comprehensive biomarker components relevant to an endless list of diseases for diagnostics, biomarker discovery and therapeutic drug monitoring [120]. SERS investigation using whole blood samples [124,125,126]; serum [46,47,127]; and plasma [128,129] dominates past and contemporary research landscape.

Human blood plasma and serum are the most studied bodily fluids for disease diagnosis, biomarker discovery and therapeutic drug monitoring. As a connective tissue circulating all over the human body, plasma composition is continuously equilibrated with the extracellular fluid of virtually every tissue compartment, providing information about cell turnover, inflammation, and antioxidant capacity [122]. In short, when these processes are perturbed in virtually any disease, plasma reflects the complete physiologic and pathologic state of the human body system, making blood tests using plasma a routine in clinical diagnosis [130]. Blood plasma composition is predominantly water (≥90%), with over 114,000 known metabolites at varying concentration levels (<1 nmol/L to 1 mmol/L), minerals, organic substances and gases [123,131,132]. Its molecular components are dominated by proteins of different molecular weight fractions (albumin, globulins, fibrinogen and a thousand others) [131,132], together with constituents like carbohydrates, lipids and amino acids. It is obvious that all the biomarkers earlier mentioned in the preceding section are present in plasma. And their fingerprint can be derived from SERS measurements, given their Raman spectrum are in the range of 400–2000 cm^−1^ wavenumbers, where bond vibrations at 470–1200 cm^−1^ are associated with carbohydrates; 980, 1080 and 1240 cm^−1^ are associated with some metabolites and nucleic acid phosphate groups; 1500–1700 cm^−1^ are associated with proteins; and higher wavenumbers (2700–3500 cm^−1^) are attributed to CH, NH, and OH stretching in protein and lipids [38].

### 4.2. Target Analytes in Biological Matrices

The target analytes in biological matrices, basically the biomarkers, include proteins, nucleic acids, metabolites/small molecules, and extracellular vesicles [6]. Any change in the respective biomarker levels, structural aspects, functional behaviour, or pharmacological actions can be correlated with initiation, progression, and regressive aspects of disorders and the body’s response to these events, which makes biomarkers vital in detecting, assessing, diagnosing, prognosticating, and monitoring various diseases/disorders [133]. The diversity of the different biomarker classes and their concentration ranges in respective biological matrices influences the choice of SERS strategies for their detection.

#### 4.2.1. Proteins

In the human body, there are more than 20,000 proteins coded for by genes, with many thousands of these found in blood (whole, plasma and serum) either through direct secretion or cell leakage [131,132]. They regulate biological processes; therefore, they can be harnessed for diagnosis and other health-related purpose based on their concentration, extent of expression, defects, and distribution [132,134,135]. A non-exhaustive list of proteins targeted in SERS assays includes peptides, enzymes, and antibodies, albumins, globulins, fibrinogen, hormones, cytokines, chemokines, adipokines and growth factors. Twenty-two proteins (serum albumin, globulins, fibrinogen and other high molecular weight fractions) account for 99% of total plasma/serum proteins, while low molecular weight fractions make up the remainder [120,123]. Valuable clinical information from this rich variety of proteins can be extracted. However, many of these disease-associated protein biomarkers are typically present in low concentrations and coexist with other high-abundance biomolecules, making their detection extremely challenging, especially from the protein corona formation associated with complex biological matrices [136,137,138,139]. In SERS measurements, different protein detection strategies are available that target a single protein biomarker or collate a panel of multiple proteins when no known biomarker is available. This is in addition to readily fabricated nanotags functionalised with different biorecognition elements for multiplex protein detection. Hence, SERS is a viable technique for detecting different types of protein-based biomarkers using single or multiplex formats [39,40,132,140].

#### 4.2.2. Nucleic Acids

Nucleic acid biomarkers, e.g., DNA, RNA, and their variants, are relevant in biomedical analyses, given that changes such as the abnormal expression levels of some miRNAs, point mutations in DNA sequences, and altered levels of DNA methylation are significant evidence of a disorder [141]. For example, tumour-related biomolecules (circulating tumour DNA, ctDNA; circulating free DNA, cfDNA mutated fragments) enter the bloodstream during cancer apoptosis, generating a broad set of cell-free biomarkers valuable for early-stage detection, prognosis and neoplasm monitoring [134]. Because nucleic acids of interest (miRNA, ctDNA, etc.) are normally in very low concentration, PCR and other amplification assays are often preferred; however, the region of interest must be known for these assays to be designed appropriately [142]. This supports the need for nucleic acid detection by SERS, alone or in combination with amplification techniques such as PCR, with multiple approaches and modalities developed [134,143]. SERS sensitivity can detect nucleotides at very low concentrations, with substrate engineering enabling reproducible spectra [45]. The multiplexing capability of SERS means it can simultaneously detect multiple nucleic acid biomarkers, which is important for point-of-care (POC) detection, high-throughput screening, and many other forms of biomedical research [71]. Among the SERS-based nucleic acid approaches, assays with a base-pairing configuration are common, whereby a probe sequence complementary to the target DNA sequence of interest is labelled with a fluorescent dye, allowing detection of a specific target sequence [40]. Another approach is to functionalise NPs with DNA sequences specific to the target biomarker [40]. Several studies have fabricated SERS substrates for detecting DNA [52,144,145] and RNA [146,147,148] in biological matrices.

#### 4.2.3. Metabolites/Small Molecules

There are more than 100,000 metabolites at varying concentrations in blood-based samples [123]. They are expressed regularly during metabolic processes; hence, a deviation in expected concentration (increase/decrease), location, and physicochemical properties can be a result of a health disorder, which is useful in clinical diagnosis [36]. At reduced matrix interference, the metabolome with Raman cross-sections is directly accessible to SERS substrates, with diverse Au/Ag substrates fabricated for the detection of clinically relevant metabolites; for example, Chu Yuhan et al. utilised a two-step enhancement strategy with synthesised AgNP colloids in a SERS-based toxicological screening of three special drugs with potential high dosage toxicity, namely clozapine, isoniazid, and carbamazepine in blood, urine and breast milk [149]. SERS have been used in related assays for the detection of several other metabolites—such as glucose [105,109,150]; urea/uric acid [151,152]; creatinine [104]; and drugs [22,153,154]—in biological matrices.

#### 4.2.4. Extracellular Vesicles (EVs)

Extracellular vesicles (EVs) are important biological messengers involved in cell-to-cell communication. They carry packages of select nucleic acids, proteins and lipids from their parent cells and hence have diagnostic importance across various diseases [36,155]. For example, exosomes produced in the endosomal compartment and secreted into body fluids contain both membrane and cytosolic information about the originating parent cells and their microenvironment, which makes EVs a viable biomarker [134,155]. In addition to techniques for detecting EVs, such as flow cytometry, Western blot, and direct fluorescence imaging, SERS is being adapted for detecting EVs, where the detection exploits either the intrinsic molecular fingerprint of the vesicle membrane by direct SERS or antibody-mediated capture of specific surface proteins by SERS nanotag immunoassay [118,156,157,158].

### 4.3. Blood Plasma and Matrix-Derived Challenges

The principal limitation of the label-free approach in plasma analysis is the susceptibility of spectral quality to interference from the matrix itself. These challenges can be classified into four, namely, the protein corona, spectral interference, nanoparticle instability and degradation, and pre-analytical variability.

The introduction of NPs into a plasma-containing medium results in rapid adsorption of plasma protein onto the NP surface, forming a protein corona that alters the NPs’ physicochemical properties and influences NP behaviour in plasma. Based on kinetics, the hard corona with higher adsorption affinity forms an inner layer of slow-dissociating proteins, while the soft corona comprised of proteins with lower binding affinities forms the outer layer that is in constant flux [137,139]. The formation of the protein corona has three analytically catastrophic consequences for SERS. First, it physically displaces analyte molecules from the plasmonic hot-spot region at the nanoparticle surface, reducing their proximity to the maximum near-field enhancement zone and thus suppressing their SERS signal. Secondly, the corona proteins themselves, particularly albumin, fibrinogen, and other high-abundance protein fractions, also generate SERS signals when they adsorb directly onto the NP surface. And the intensity of these background signals can sometimes mask the signals of low-abundance target biomarkers. Thirdly, the corona alters the nanoparticle surface charge, reducing electrostatic repulsion and promoting nanoparticle aggregation with consequent loss of SERS reproducibility. The protein corona composition is not static and is constantly evolving based on the Vroman effect, in which initially adsorbed high-abundance proteins are progressively displaced by lower-abundance proteins of higher affinity as the equilibration proceeds. This Vroman effect introduces spectral drift during SERS measurements performed at different timeframes, leading to significant measurement irreproducibility in plasma SERS protocols.

The second challenge, spectral interference in plasma SERS, arises when molecular components of the matrix generate Raman bands that overlap with, mask, or are misidentified as the target analyte signal. Unlike fluorescence background, which can be partially removed by baseline correction algorithms, spectral interference from structurally defined plasma molecules produces sharp, reproducible Raman bands at specific wavenumber positions that are indistinguishable from genuine analyte signals unless their molecular origin is known. Albumin is the dominant spectral interferent in plasma SERS, generating intense bands that coincide with diagnostically important regions of the SERS spectrum for other target analytes [123,137]. Also, haemoglobin released from lysed erythrocytes in haemolytic plasma specimens generates several intense Raman bands arising from its porphyrin ring and iron–histidine coordination environment. These bands can dominate the SERS spectrum and saturate the CCD detector, rendering the measurement analytically valueless. Another interferant, lipoproteins, contributes a characteristic band that overlaps with carbohydrate and nitrogenous bases in the same region.

The third challenge is the colloidal stability of SERS nanoparticles in plasma when NPs are critically compromised by the high ionic strength of the plasma environment. Citrate-stabilised AuNPs suspensions, which rely on electrostatic repulsion between negatively charged surface ligands to prevent aggregation, are stable in deionised water but undergo rapid aggregation in physiological saline where the Debye screening length becomes insufficient to maintain an electrostatic repulsion energy barrier against the van der Waals attractive force between particles. This critical aggregation produces highly variable hot-spot distributions whose SERS signal intensities fluctuate between measurements, eliminating any possibility of quantitative calibration. For AgNPs, an additional metal-specific degradation mechanism occurs in plasma. Oxygen and plasma constituents that contain sulphur react with the Ag surface to form SERS-inactive compounds. The SERS activity of non-passivated AgNPs becomes rapidly extinguished in plasma, thereby confounding SERS measurements [136,139].

Lastly, pre-analytical variability, where variation in plasma composition arises from sample collection, processing, and storage conditions rather than from genuine biological differences between subjects, is the most often overlooked source of irreproducibility in plasma SERS measurements. The sensitivity of SERS is such that alterations in the molecular composition of the plasma matrix seriously affect the SERS spectral profile. Also, the choice of anticoagulant affects plasma SERS spectra. EDTA (ethylenediaminetetraacetic acid), heparin, and citrate anticoagulants are the three most commonly used agents in clinical plasma collection, and each introduces unique compositional changes. EDTA chelates divalent cations (Ca^2+^, Mg^2+^), altering the protein conformation of calcium-dependent plasma proteins and changing their nanoparticle adsorption behaviour. The EDTA molecule also generates SERS signals when they adsorb onto Au and Ag NP surfaces. Heparin binds to several plasma proteins and alters the protein corona composition relative to EDTA or citrate plasma. Plasma can be diluted by the citrate anticoagulant present in blood collection tubes, hence reducing all analyte concentrations and altering electrolyte balance. Repeated freeze–thaw cycles induce protein denaturation and aggregation in plasma, alter lipoprotein structure, and release intracellular contents from intact residual cells or platelet fragments in the plasma sample [159]. The cumulative structural alteration in plasma due to repeated freeze–thaw cycles can cause significant spectral shift during SERS measurements. Processing delay between the time of blood collection and plasma processing allows ongoing metabolic activity of residual leukocytes and platelets in whole blood, which alters glucose, lactate, and cytokine concentrations in a time-dependent manner. These pre-analytical variables are a primary cause of false-positive and false-negative classifications.

The four challenges identified above often reinforce each other; for example, protein corona formation promotes NP aggregation and introduces spectral interference through adsorbed protein SERS bands simultaneously. Similarly, NP aggregation creates variable hotspot distributions that amplify the impact of any residual spectral interference, while pre-analytical variability alters the protein corona composition, the degree of nanoparticle aggregation, and the intensity of spectral interference in a sample-specific manner.

Effective plasma sample preparation for SERS therefore requires a multi-step, integrated approach that addresses all four challenge domains in a defined sequence: first, pre-analytical standardisation to control the composition of the starting plasma; second, matrix simplification to reduce the concentration of dominant interferent proteins and stabilise the nanoparticle system; third, nanoparticle surface engineering to resist corona formation and degradation; and fourth, measurement standardisation to compensate for residual inter-sample variation [160,161]. In response, these matrix-derived challenges have motivated the development of sample pretreatment strategies, including molecular weight filtration, pH adjustment, and dilution, intended to selectively reduce HMWF interference without substantial loss of diagnostically relevant spectral information [39,86,162]. Their comparative performance is examined in Section 5 in the context of specific diagnostic applications.

## 5. SERS, Plasma and AuNP/AgNP Nanostructures Utility in Disease Diagnostics

In this section, the SERS detection strategies are organised based on signal transduction mechanisms and platform complexities into (i) single-mode detection, wherein SERS is the only transduction mechanism, whether through label-free spectral fingerprinting or targeted indirect immunoassay; and (ii) multi-mode detection, in which SERS is combined with other independent transduction platforms such as electrochemical, thermoplasmonic, or colourimetric sensing that enables cross-validated diagnostic readouts. This framework is complementary to the conventional direct (label-free) and indirect (labelled/tagged) classification introduced in Section 2. Also, the single-mode detection classification is further differentiated by substrate type (colloidal or engineered solid support) and by direct/indirect detection strategy. This progression from label-free SERS using simple colloidal assays to multiplex and multimodal platforms presented in this section aims to provide a coherent structure that reflects the practical exploration of plasma-based SERS research for disease diagnostics.

### 5.1. Single-Mode Single/Multiplex Analyte Detection

The direct interaction of blood plasma components with AgNP/AuNP substrates to generate spectral fingerprints for disease detection is the basis of label-free SERS, which is particularly suited to diseases lacking specific biomarkers and for profiling a disease associated with multiple biomarkers [42,45]. However, a major limitation resulting from matrix interference by plasma HMWF has necessitated diverse assays exploring simple colloidal suspension, anisotropic NP synthesis, fabricated substrate support, and advanced chemometric algorithms to minimise matrix interference on SERS diagnostic performance [162].

#### 5.1.1. Colloidal Substrate Strategies in Oncological Diagnostics

In the detection and differentiation of neoplasms, colloidal suspensions of Au and Ag NP substrates are very popular. When colloidal plasmonic NPs interact with the constituents in plasma samples, a spontaneous aggregation is induced that increases hotspot densities with attendant signal enhancement. Although the aggregation occurs at random, the interpretation of resulting SERS spectra using chemometrics has consistently yielded useful results across a range of oncological conditions.

This approach was exemplified in the detection of colorectal cancer (CRC) and its precursor adenomatous polyps (AP) from blood plasma. By using as-synthesised spherical AgNP colloids incubated in a 1:1 ratio with plasma, ten common Raman bands were identified across CRC, AP, and healthy control (HC) groups, with four bands at 725, 892, 1004, and 1368 cm^−1^ showing elevated intensities in CRC. Characteristic band shifts at wavenumbers 892 to 902 cm^−1^ and 1368 to 1349 cm^−1^ in CRC (absent in HC and AP) were identified as diagnostically significant. Partial least squares with linear discriminant analysis (PLS-LDA) returned areas under the receiver operating characteristic curve (AUC) of 0.938, 0.869, and 0.945 when comparing the three groups against each other, with an overall diagnostic sensitivity of 86.4% and specificity of 80.0%. This performance compares favourably with conventional faecal immunochemical and occult blood tests, which had specificities ≥ 95%, but their sensitivities ranged from 65.8% to 81.8% for CRC and 27.1% to 41.3% for advanced adenomas [87]. 

The same research group also applied label-free AgNP colloids to cervical cancer (CC) classification, profiling plasma from 60 CC patients and 50 healthy controls [163].An empirical algorithm based on integrated spectral areas yielded an 83% sensitivity and a 78% specificity. However, by using principal component analysis combined with linear discriminant analysis (PCA-LDA), a 96.7% sensitivity, a 92% specificity, and an AUC of 0.994 were returned, which was a significant (15%) improvement in sensitivity over the empirical approach, demonstrating the importance of advanced chemometrics in label-free SERS [161]. Notably, the SERS band at regions around wavenumbers 632 cm^−1^, 725 cm^−1^, and 1655 cm^−1^, previously identified in both the colorectal cancer and the adenomatous polyps plasma SERS spectra, also featured in the CC spectra, which might imply a possible significance in neoplastic SERS profiles.

Extending PCA-LDA to nasopharyngeal cancer (NPC) staging, but using a colloidal AuNP platform, Lin Duo and colleagues demonstrated the capacity of plasma SERS to discriminate not only between healthy individuals and cancer patients but also between early (T1) and advanced (T2–T4) tumour stages [164]. PCA-LDA analysis of SERS spectra from AuNP-plasma mixtures yielded diagnostic accuracies of 83.5% for T1 in contrast to HC and 93.3% for T2–T4 in contrast to HC, with AUC values of 0.955 and 0.981 for the respective comparisons, supporting a strong potential for differentiating HC from NPC plasma samples. Remarkably, inter-stage discrimination between T1 and T2–T4 yielded an AUC of 0.641, significantly lower than outcomes for HC comparisons, hence highlighting a limitation of distinguishing early-stage NPC from more advanced NPC within a single spectral framework [164].

A methodological departure from the traditional statistical PCA-LDA technique to the Artificial Intelligence (AI)/Machine learning (ML) algorithm was demonstrated in the classification of acute myeloid leukaemia (AML) subtypes (M3, M5, and other oAML) from healthy controls [10]. Following a short incubation (15 min, 4 °C) of AgNP colloidal suspension with plasma, and utilising both the band intensity data and whole spectra data in the classification, PCA combined with classification and regression tree (CART) algorithm, an AI-based predictive algorithm, returned an overall classification accuracy of 89.8% for AML versus HC, with AUC values of 0.955, 0.968, 0.934, and 0.707 for the respective HC vs. AML, M3 vs. M5, M3 vs. oAML, and M5 vs. oAML pairs. The short incubation time and the large validation cohort (222 plasma samples) represented improvements in assay throughput, and employing CART enhanced statistical robustness [10].

Another ML/AI-based Gaussian kernel support vector machine (SVM) classifier, excellent for handling complex data, was used in comparing the diagnostic performance of Raman spectroscopy with SERS in discriminating ovarian cancer (OvC) from benign gynaecological conditions (as control, HC) [11]. Both Raman and SERS measurements for respective plasma samples mixed with spherical AgNP colloids generated corresponding spectra with five characteristic Raman bands identified as significant. Counterintuitively, SVM classification between OvC and HC for Raman spectroscopy achieved superior classification performance (94% sensitivity, 96% specificity) compared to SERS (87% sensitivity, 89% specificity). Generally, SERS, due to the signal-enhancing properties of its plasmonic substrate, is acknowledged as being more sensitive than Raman spectroscopy, especially in complex matrices such as plasma. However, this report provides interesting possibilities for further investigation. The age difference of the sample population was accounted for, alongside laboratory screening to exclude the possible impact of plasma CA-125 levels (an upregulated cancer biomarker) in all the samples prior to usage in the SERS experiment, which provided a valid parameter for comparing results. Hence, after excluding age and CA-125 as confounding factors, the presence of the anticoagulant (EDTA) in the plasma samples is a plausible reason for the reduced accuracy of SERS measurements. Also, the protein corona from HMWF plasma proteins might have impacted the results [134,136]. It could also be that changes in the experimental setup, together with AI data interpretation methods such as SVM, can improve results from Raman spectroscopy significantly.

**Figure 7:**
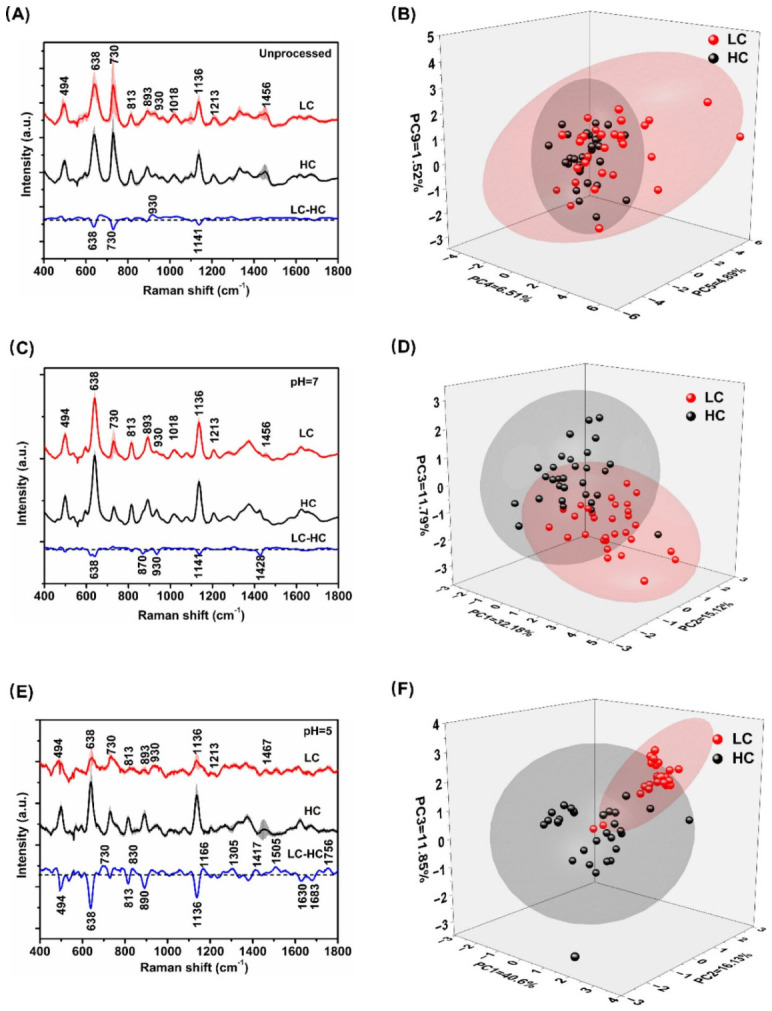
Mean and difference spectra of early-stage LC and HC for unprocessed plasma (**A**), plasma at pH 7 (**C**), and plasma at pH 5 (**E**). Three-dimensional PCA score plots for unprocessed plasma (**B**), plasma at pH 7 (**D**), and plasma at pH 5 (**F**). [Source: Reprinted with permission from Lu Dechan et al., 2025 [86]. 2025 American Chemical Society].

SERS assays are sensitive to matrix interference from plasma HMWF protein, which affects result reproducibility, thus necessitating a variety of sample pretreatment strategies to mitigate. A simple, low-cost pretreatment protocol involving a tenfold dilution of plasma samples combined with sample pH adjustment to 5 was evaluated for its capacity to improve SERS signal consistency and classification accuracy in lung cancer (LC) detection by Lu Dechan and colleagues [86]. Droplets from a mixture of equal-volume spherical AgNP colloidal suspensions with pretreated plasma samples (AgNP@Plasma) were covered with liquid paraffin to prevent the coffee-ring effect due to evaporation. This allowed SERS measurements in both the wet and dry states. The pH 5 samples were benchmarked against untreated plasma and plasma samples adjusted to pH 7. The mean and difference SERS spectra of LC and healthy control (HC) samples under each pretreatment condition are shown in Figure 7A,C,E, with corresponding three-dimensional PCA score plots in Figure 7B,D,F. Across all pretreatment conditions, LC and HC samples shared ten characteristic Raman bands, with a marked difference at wavenumbers 638 and 730 cm^−1^ respectively attributed to tyrosine and hypoxanthine/adenine vibrations being more pronounced in the pH 5 samples (Figure 7A,C,E), an indication that slightly acidic conditions might favour selective adsorption of diagnostically relevant low-molecular-weight (LMW) plasma constituents onto the AgNP surface, while partially suppressing the dominant protein corona. The wet measurement (with paraffin) showed no coffee rings and yielded a relative standard deviation (RSD) of 14.04% compared to 22.3% for unshielded samples (without paraffin), demonstrating improved spectral reproducibility, although with an attendant decrease in band intensity. PCA-LDA classification applied to adjusted pH samples data returned 93.3% sensitivity and 90.0% specificity, with an AUC of 0.975, representing a substantial improvement over the classification of unprocessed plasma. A noticeable increase in the PCA score plots and inter-group separation progressed from unprocessed plasma (Figure 7B) through pH 7 samples (Figure 7D) and then to pH 5 samples (Figure 7F), suggesting that even minimal sample pretreatment can influence the results in label-free SERS. This has practical implications, since neither filtration equipment nor substrate derivatisation is required, making this simple strategy viable for resource-limited or point-of-care implementations pending validation in larger clinical cohorts.

**Figure 8:**
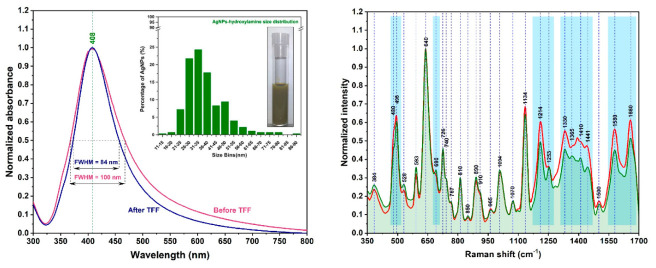
(**Left**) Superposition of the normalised UV-VIS absorption spectra of AgHya colloid recorded before (red curve) and after (blue curve) TFF purification. Inset: photograph of the purified colloid and size histogram. (**Right**) Overlapping of the mean SERS spectra recorded on blood plasma samples collected from HC (green spectrum) and BC patients (red spectrum) recorded on SSPSs using a 785 nm laser. Spectra were normalised to the 640 cm^−1^ peak. [Source: Reprinted from Știufiuc et al., [159] MDPI Creative Commons License (CC-BY)].

Polydispersity in nanoparticle size distribution, chemical residues from synthesis, and uneven clustering behaviour on plasmonic NPs in contact with plasma constituents are among the substrate-derived sources of spectral variability and result irreproducibility in colloidal SERS assays. Diverging from sample pretreatment options, Stiufiuc and colleagues investigated whether improvements in NP monodispersity could enhance the diagnostic classification of breast cancer (BC) from blood plasma [165]. They addressed substrate variability through tangential flow filtration (TFF) of synthesised AgNP colloids. This resulted in a reduction of NP diameter from 100 nm to 84 nm and narrowed the full width at half-maximum (FWHM) of the UV-Vis absorption band by 16%, as shown in Figure 8 (Left), with the inset showing the associated narrowing of the particle size distribution. The processing step achieved high inter-batch reproducibility and around 3 × 10^3^ EF. A SERS substrate formed by depositing the TFF-processed AgNP on a CaF_2_ glass ensured consistent SERS measurements, enabling the label-free discrimination of 29 breast cancer (BC) samples from 35 healthy controls (HC). Similarities in SERS spectral bands in both HC and BC samples are shown in Figure 8 (Right), with wavenumber differences from the superimposed spectra highlighted in blue. The subtle spectral variations encode relevant diagnostic information interpreted through multivariate analysis, with PCA-LDA classification achieving 90% sensitivity, 89% specificity, and 89% overall accuracy between HC and BC. In comparison, mammography (the BC screening gold standard) achieves approximately 70% sensitivity and 75% specificity. Hotspot variability due to substrate heterogeneity is a major source of intra-batch variability in SERS results; hence, any process that maximises monodispersity is a promising option. It is apparent that post-synthesis NP purification can reduce polydispersity without the need for complex synthesis, elaborate solid-substrate design, or extensive sample pretreatment. This makes the TFF-optimised SERS assay clinically promising as a diagnostic method to enhance SERS result reliability and reproducibility, once a larger clinical cohort validates these findings.

#### 5.1.2. Solid-Support and Anisotropic Substrate Strategies

As already mentioned, the random aggregation of colloidal NPs in plasma and other biological matrices introduce batch-to-batch SERS result variability and restricts quantitative reproducibility. Strategies employed to address this challenge include selective fabrication of solid-support substrates and utilising anisotropic/anisometric NPs. These options exploit spatially defined hotspot distributions and geometry-driven enhancement fields that are not dependent on liquid-based aggregation dynamics.

AgNP nanostars are known to exhibit higher hotspot density per isolated particle due to the “lightning rod effect” from sharp tips in multiple branched arms on a tiny core, which also produces SERS enhancement two or more orders of magnitude stronger than non-aggregated plasmonic nanospheres [68].This shape-induced SERS enhancement was explored by Freitas and colleagues in their use of NP nanostars for stroke detection in plasma, aimed at rapid pre-hospital diagnoses and faster treatment intervention [17]. By exploiting the superior EF from Ag and the high hotspot densities from anisotropic geometries, citrate-capped colloidal AgNPs (stars) detected levels of glial fibrillary acidic protein (GFAP), an associated haemorrhagic stroke biomarker in plasma at clinically relevant concentrations. The deployment of Light Gradient Boosting Machine (LightGBM), a machine learning (ML) model for classification of the resulting SERS spectra, achieved a predictive accuracy of 83% for differentiating stroke subtypes. The use of readily synthesised anisotropic substrates represents a promising method to circumvent aggregation-based challenges when using colloidal suspensions, and the combination of the AI/ML algorithm in SERS data interpretation further supported the potential of this developed platform for rapid pre-hospital emergencies [17]. The use of a Raman-specific regularisation algorithm, such as the Logistic Regression with Peak-Sensitive Elastic-Net Regularisation (PSE-LR) algorithm, in place of LightGBM could be a more viable choice of AI model, given it is specifically suited to interpreting SERS and related spectroscopic datasets.

Present advances in fabrication technologies have resulted in more precise SERS substrate design and fabrication that enable custom SERS platforms to be developed for direct pathogenic microorganism detection. This was demonstrated using a proprietary polymer spinning technique to produce a two-layer polymer mat with predetermined nanoscale pores and a physical vapour deposition (VPD) to coat the mat with Au:Ag alloy NPs to form SERS substrates for the detection of *Staphylococcus aureus* (*S. aureus*), *Pseudomonas aeruginosa* (*P. aeruginosa*), and *Salmonella Typhimurium* (*S. Typhimurium*) in blood plasma [166]. Notably, the Au:Ag@polymer simultaneously served as an SERS substrate and a high-throughput filter that can entrap microbial cells contained in biofluids, thereby bypassing the need for filters in sample pretreatment. SERS measurements in the three pathogenic plasma samples showed shared bands at 730, 782, 1034, 1100, 1330 and 1452 cm^−1^. Compared to the others, *P. aeruginosa* had very weak bands at 726 and 1330 cm^−1^, distinct low-intensity bands at 850, 885, 1205 and 1306 cm^−1^ and a highly intense band at 675 cm^−1^. For *S. Typhimurium*, characteristic bands were at 650, 958, 1273 and 1376 cm^−1^. The intense band at 730 cm^−1^ was significant for *S. aureus*, with other distinct bands at 956, 1244, and 1403 cm^−1^. The reproducibility of the SERS signals using the reusable Au:Ag@polymer substrate gave a relative standard deviation (RSD) of 8% when calibrated to the intense band at 730 cm^−1^, and 12% for the weak band at 958 cm^−1^, demonstrating acceptable reproducibility for a solid-support fabricated substrate. A sharp distinction in the spectra profiles from pristine plasma and respective pathogen-containing plasma enabled visual identification of respective pathogens without the need for advanced chemometrics. Also, the tuneability of the polymer mat pore dimensions hold promise in fabricating custom SERS substrates with both filtering and reproducible signal enhancement capacity [166].

#### 5.1.3. Metabolic and Non-Oncological Targets

In addition to cancer classification, label-free SERS has been utilised to detect metabolic disorders and pharmaceutical compounds in blood plasma. Because these non-oncological conditions induce widespread, systemic biochemical alterations, they are uniquely suited to the holistic molecular mapping provided by global spectral profiling.

When colloidal SERS substrates are used in SERS measurements, the spontaneous and random aggregations of plasmonic nanoparticles add to the variability of generated SERS spectra. SERS substrates on solid support are fabricated to allow optimal enhancements without inducing aggregation. To minimise random aggregation, the combination of Langmuir–Blodgett (LB) deposition and self-assembly techniques was used in a SERS assay by the Das team [167] to discriminate blood glucose levels directly in healthy, pre-diabetic, and diabetic blood plasma. The fabrication of 4-cyano-4’-pentylbiphenyl (5-CB) liquid crystal Langmuir–Reverse Schaefer (L-RSh) films by automated deposition, followed by the self-assembly embedding of colloidal AuNP (spheres) on the film surface via submerging in AuNP colloidal solution, formed the SERS substrate. Sample deposition was achieved by spin-coating plasma over the substrate to get a uniform spread, and 4-mercaptopyridine (4-Mpy) was used as the calibration standard. Potential fingerprint Raman bands from the SERS spectra were at 630, 725, 953, 1143, 1145, and 1003 cm^−1^, which became intense with increasing glucose content; branched-chain amino acid (BCAA) bands were at 910, 1259, and 1296 cm^−1^. An intense band at 1361 cm^−1^ (in pre-diabetic samples) shifted slightly to 1365 cm^−1^ (in diabetic samples) and was attributed to glucose molecules, which was corroborated by glucose spiking. The analytical performance via PCA and LDA multivariate data analyses returned 100% accuracy for cross-validated grouping and a 75.8% combined group accuracy, with AUCs of 1 (healthy/pre-diabetic), 0.983 (healthy and diabetic), and 0.868 (pre-diabetic/diabetic). This indicates potential use in blood glucose monitoring, capable of discriminating between normal, high (pre-diabetic), and extreme (diabetic) blood glucose levels in plasma [167]. A remarkable aspect here is that the introduction of computer-assisted film deposition provides a surface that enhances self-assembly monolayer formation with optimal hotspot densities, alongside dense AuNP clusters that reduce the elastic distortions by coalescing. Spin coating samples creates a uniform spread of blood plasma on the surface, minimising both the coffee-ring effect of colloidal methods and protein corona masking, given that more plasma components can interact with the enhanced field of the substrate. 

### 5.2. Single-Mode Detection: Labelled and Indirect Approaches

Indirect (labelled/tagged) SERS detection, in which the analytical signal originates from reporter molecules conjugated to plasmonic NP substrates rather than from the analyte itself, enables selectivity through biorecognition-mediated capture. The incorporation of antibodies, aptamers, or other ligands as targeting elements enables specific analyte binding from the complex plasma matrix, while the narrow and customisable spectral signatures of Raman reporters support multiplexed detection of multiple targets within a single assay. The principal formats employed, most often sandwich immunoassays, aptamer-based sensing (aptasensors), and hybrids linked with internal standards, are presented below, organised from basic single-analyte configurations to multiplexed and methodologically hybrid platforms.

#### 5.2.1. Single-Analyte Sandwich Immunoassays

The sandwich immunoassay format, in which an antigen is captured between two antibodies, where one antibody is immobilised on a solid substrate and the other antibody is conjugated to a SERS-active nanotag, is among the most prevalent labelled SERS assay formats. This architecture provides a high degree of analyte specificity while the embedded reporter molecule generates a stable, quantifiable SERS signal proportional to analyte concentration [56].

**Figure 9:**
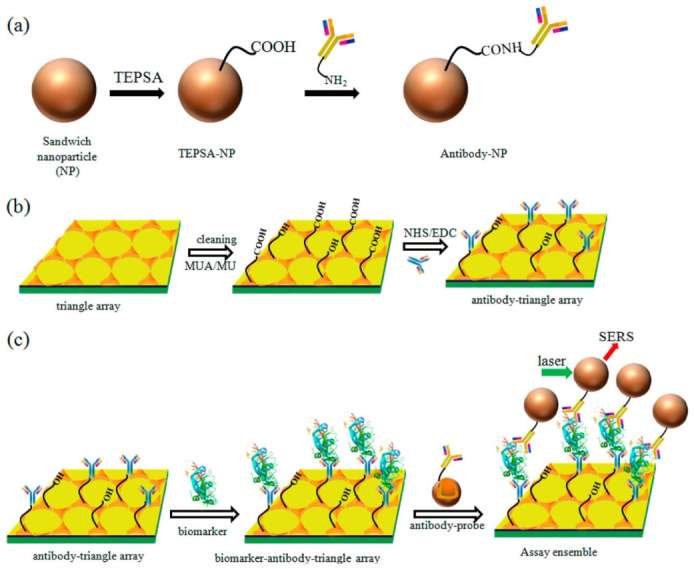
Schematic illustration of conjugation of the (**a**) SERS probe (sandwich nanoparticle) to the detection antibody and the (**b**) Au triangle nanoarray chip to the capture antibody. (**c**) Schematic illustration of the operating principle of the SERS immunosensor for biomarker detection. [Source: Reprinted with permission from Li Ming et al., 2013 [168]. 2013 American Chemical Society].

The transition from label-free colloidal SERS to structured sandwich immunoassays introduces a layer of analytical specificity that permits quantitative detection of individual protein biomarkers at clinically relevant concentrations directly from plasma. Two reported immunosensor configurations, one targeting vascular endothelial growth factor (VEGF) and the other carcinoembryonic antigen (CEA), illustrate contrasting strategies for generating and amplifying biomarker-dependent SERS signals, respectively, and are represented schematically in Figure 9. Although developed independently, both platforms exemplify the sandwich immunoassay.

The VEGF immunosensor developed by Li Ming and colleagues [168] exploited the coupling of two anisotropic plasmonic architectures to generate a three-dimensional (3D) enhancement field capable of detecting VEGF_165_ at picogram concentrations in breast cancer (BC) plasma samples. The detection probe was assembled from AuNP nanostars functionalised with malachite green isothiocyanate (MGITC) as a Raman reporter, encapsulated in a silica shell and then conjugated to a VEGF polyclonal antibody. A separate capture platform comprising a gold triangle nanoarray chip produced by nanolithography and functionalised with VEGF monoclonal antibody (containing two binding sites) completed the sandwich configuration (Figure 9a–c). Detection occurs when VEGF present in the plasma bridges the two antibody-functionalised components, linking the triangular nanoarray edges to the nanostar tips under laser excitation. The resulting near-field coupling between these two geometrically compatible anisotropic components (with high hotspot densities) generates a 3D plasmonic enhancement that amplifies the MGITC reporter signal at 1578 cm^−1^, enabling quantification within the 301–588.8 pg mL^−1^ range. The reported sensitivity was comparable to standard ELISA (337–601 pg mL^−1^) and was conducted with a marked advantage in detection speed. Rather than relying on a single nanoparticle surface for enhancement, the immunosensor converts a biorecognition event into a constructive plasmonic coupling between two matched anisotropic nanostructures, a strategy that transforms analyte binding directly into measurable field amplification. This makes the configuration analytically significant.

A structurally distinct but functionally analogous sandwich immunosensor was developed for carcinoembryonic antigen (CEA), a 200 kDa glycoprotein upregulated across multiple carcinomas by Lu Dechan and colleagues [12]. This complementary sandwich format was based on a bimetallic core–shell detection probe and a boronic acid-functionalised capture substrate. The detection probe (Au@4MBN@Ag NP-Ab) combined an AuNP core, a 4-sulfanylbenzonitrile (4MBN) Raman reporter, an AgNP shell for enhanced signal generation, and a CEA-specific antibody. The capture substrate was a two-dimensional (2D) platform functionalised with 4-mercaptophenylboronic acid (4-MPBA), whose boronic acid groups provide reversible covalent affinity for glycoprotein targets through selective binding to cis-diol moieties. The composition of the capture substrate supplements antibody-mediated capture with an additional layer of selectivity specific to glycosylated cancer biomarkers such as CEA. The 4MPBA Raman band at wavenumber 999 cm^−1^ was used in calibration, with the 4MBN reporter band at 2226 cm^−1^ increasing proportionally with CEA concentration across a linear range of 10^−9^ to 10^−11^ M in plasma, with an LOD of 5.3 × 10^−16^ M. Remarkably, the stability and intensity of the reporter signals were maintained in the presence of five additional proteins, indicative of analytical specificity in plasma where competitive adsorption from other constituents affects results. This resistance to cross-reactivity is important, especially when the formation of a protein corona is a challenge encountered in SERS assays. The choice of a boronic acid reporter complements the specificity of the antibody, thus ensuring stable signals.

Together, these two immunoassay configurations, the VEGF format exploiting geometric plasmonic coupling between anisotropic structures, and the CEA format that combined chemical and biological recognition, both demonstrated that rationally designed SERS immunosensors can deliver diagnostic performance from plasma samples comparable to ELISA without sacrificing analytical specificity. Translational progress for both platforms depends on validation in demographically diverse and larger clinical cohorts involving real patient samples.

#### 5.2.2. Multiplex Labelled Detection

A key advantage of indirect SERS over fluorescence-based immunoassays is the narrow full width at half-maximum (FWHM) of Raman reporter bands, which permits multiple spectrally resolved signals to be acquired simultaneously under a single excitation wavelength. This property has been exploited to develop highly multiplexed labelled SERS platforms for the simultaneous quantification of several plasma biomarkers.

Simultaneous detection of multiple inflammatory biomarkers in a single plasma sample requires strict signal specificity, cross-reactivity management, and substrate uniformity that challenge single-mode SERS platforms. Kamińska and colleagues addressed these requirements through the rational design of a microfluidic SERS immunoassay capable of multiplex quantification of three interleukins (IL-6, IL-8, and IL-18) in blood plasma in a single analytical workflow [78]. 

**Figure 10:**
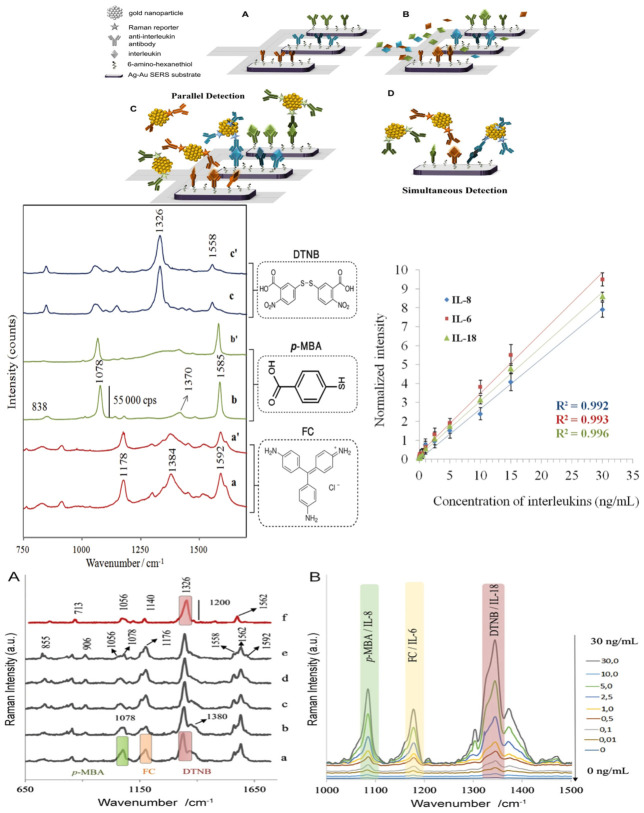
(**Top**) Sequential steps for the formation of a SERS-based multiplex immunoassay. (**A**) capturing substrate, (**B**) mixture of interleukins (IL-6, IL-8, IL-18) in human blood plasma injection, (**C**) the parallel approach, (**D**) the simultaneous multiplex configuration. (**Middle Left**). (a,b,c) SERS spectra of FC, p-MBA, and DTNB adsorbed onto AuNPs (Raman reporter-AuNPs), and (a’,b’,c’) AuNPs (antibody-Raman reporter-AuNPs), respectively. (**Middle Right**). The concentration–intensity calibration curves obtained for simultaneous multiplexed detection of three interleukins—IL-6, IL-8, and IL-18—from blood plasma samples. (**Bottom A**) SERS responses in the DA-1 chamber of microfluidic immunoassay during detection of IL-18 in human blood plasma sample according to the subsequent steps of parallel approach: (a) after the mixture of equal amounts of three kinds of Raman reporter-labelled AuNPs and (b–f) after subsequent washing by PBS buffer solution. (**Bottom B**) Typical SERS responses for increasing concentrations of target IL-6, IL-8, and IL-18 interleukins in blood plasma during a simultaneous multiplexed approach. [Source: Reprinted from Kamińska et al., 2017 [78]. 2017 Springer Nature. Creative Commons Attribution 4.0 (CC-BY-4.0)].

The platform’s architecture, illustrated in Figure 10 (Top), exploited two complementary features of the SERS technique: the narrow full width at half-maximum (FWHM) of Raman vibrational bands, which permits resolution of multiple reporters within a single spectral window, and the independently tuneable chemistry of gold nanoparticles, which allows functionalisation with distinct reporter–antibody combinations. Three detection probes were synthesised by conjugating AuNP colloids with the spectrally non-overlapping Raman reporters 5,5′-dithio-bis(2-nitrobenzoic acid) (DTNB), fuchsin (FC), and p-mercaptobenzoic acid (p-MBA) alongside antibodies specific to IL-18, IL-6, and IL-8, respectively, yielding probes with orthogonal spectroscopic identities. Capture was achieved using a bimetallic Ag-Au substrate functionalised with the corresponding anti-interleukin antibodies. Two microfluidic configurations were compared: a parallel design with three spatially separated capture chambers (Figure 10, Top C), enabling sequential analyte-specific detection; and a simultaneous design with a single unified chamber housing all three capture antibodies (Figure 10, Top D), enabling co-incubation and concurrent signal acquisition.

Representative SERS spectra confirming probe identity and antibody binding affinity are shown in Figure 10 (Middle Left), where pairs of spectra (Raman reporter on AuNP versus antibody-functionalised reporter–AuNP conjugate) illustrate the preservation of reporter signatures following bioconjugation. This is a critical validation step that establishes the absence of reporter-signal quenching by protein adsorption. Concentration–intensity calibration curves for all three interleukins under the simultaneous detection configuration are presented in Figure 10 (Middle Right), demonstrating linear responses with R^2^ values of 0.992 (IL-8), 0.993 (IL-6), and 0.996 (IL-18) across the clinically relevant range of 0–30 ng mL^−1^. The sequential SERS responses within the parallel microfluidic chamber during IL-18 detection, and the corresponding multiplexed spectra at varying interleukin concentrations in the simultaneous format, are shown in Figure 10 (Bottom A and B), confirming the independent quantifiability of each analyte signal at calibration bands of 1326 cm^−1^ (DTNB), 1078 cm^−1^ (p-MBA), and 1176 cm^−1^ (FC). The LOD from PCA spectra analyses and calibration were 2.3 pg mL^−1^ (IL-6), 6.5 pg mL^−1^ (IL-8), and 4.12 pg mL^−1^ (IL-18) in the parallel format, with a modest reduction in sensitivity in the simultaneous format (3.8, 7.5, and 5.2 pg mL^−1^, respectively), probably due to competitive antibody binding and reduced analyte incubation time [78]. These detection thresholds are clinically significant because circulating IL-6 levels in sepsis and inflammatory conditions typically range from 1 to hundreds of pg mL^−1^, placing this platform within the actionable detection window for inflammatory disease monitoring. Critically, the platform demonstrates that the multiplexing capacity of SERS is not merely a theoretical advantage but can be operationalised within a microfluidic format that preserves a linear dynamic range and independent analyte detection, thereby addressing one of the central criticisms levelled at SERS-based immunoassays relative to established multiplexed immunochemical platforms.

#### 5.2.3. Combined Label-Free Profiling with Internal Standards: A Methodological Hybrid

The integration of an internal standard Raman reporter into label-free SERS substrate represents a hybrid strategy that retains the holistic plasma fingerprinting capability of direct spectral profiling while introducing a spectral calibration reference that corrects for substrate-derived signal heterogeneity. This approach bridges the conceptual divide between label-free and labelled strategies and warrants explicit recognition as a distinct methodological advance.

Chen and colleagues [121] fabricated two complementary substrates for the detection of colorectal cancer (CRC) in healthy and CRC plasma samples within an hour of sample incubation. The first substrate was a bare AgNP-on-bacterial-cellulose support (BC@AgNP), while the second was an equivalent substrate incorporating 4-mercaptopyridine (4-MP) as an internal standard (BC@4-MP@AgNP).

Four machine learning (ML) algorithms—Decision Trees (DT), Random Forests (RF), k-Nearest Neighbours (KNN), and Support Vector Machines (SVMs)—were utilised for statistical model training to evaluate generated Raman and SERS spectra. The introduction of 4-mercaptopyridine (4-MP) as an internal standard for spectral calibration enhanced the classification accuracy of the ML algorithms, producing measurable improvements in ML classification accuracy; SVM accuracy increased from 98.75% (uncalibrated) to 100% (calibrated), KNN from 95% to 97.5%, and DT from 83.75% to 86.25%. AUC values showed corresponding improvements, with KNN rising from 0.99 to 1.00 and DT from 0.84 to 0.93 following calibration. Notably, the spectral bands identified as most diagnostically relevant, including tyrosine (633 cm^−1^), hypoxanthine/adenine (720 cm^−1^), and phenylalanine (1003 cm^−1^), were consistent with those identified in independent colloidal AgNP assays for CRC and adenomatous polyps, although with a shift at 892 (902 cm^−1^) and at 1368 (1349 cm^−1^) for CRC samples [87], providing cross-platform validation of the biological specificity of these spectral features [121]. The findings highlight the value of internal standard calibration as a practical, implementable strategy for improving the reproducibility and clinical reliability of label-free plasma SERS assays.

### 5.3. Multimode Detection Strategies

The inherent complexity of blood plasma, characterised by high protein abundance, electrolyte-driven nanoparticle aggregation, and competitive molecular adsorption at substrate surfaces, creates conditions in which any single analytical transduction mechanism is susceptible to false-positive or false-negative results arising from matrix-specific confounders [9,162]. Multimode detection strategies integrate SERS with one or more orthogonal transduction platforms to provide independent analytical readouts from the same sample, enabling cross-validation of results and substantially reducing diagnostic error rates [69,169,170]. This approach is particularly pertinent in plasma-based diagnostics, where the stakes of analytical misclassification are high and where the matrix complexity that limits single-mode assays provides the greatest motivation for complementary measurement strategies. The examples reviewed below are organised by the nature of the orthogonal transduction modality combined with SERS, progressing from dual-mode optical/thermoplasmonic platforms to electrochemical–acoustic combinations.

#### 5.3.1. SERS Integrated with Thermoplasmonic Detection: Cardiac Troponin I

The thermoplasmonic (photothermal) biosensing principle relies on localised surface plasmon resonance (LSPR) to generate heat upon photon absorption, producing an optical or thermal readout that correlates with the quantity of analyte bound to a plasmonic substrate [171]. Campu and colleagues [95] exploited this principle alongside SERS for real-time microfluidic detection of cardiac troponin I (cTnI), a gold-standard biomarker for acute myocardial infarction (AMI), in blood plasma [84]. A plasmonic nanoplatform based on immobilised gold nanobipyramids was fabricated by deposition on glass slides and bonded to a microfluidic frame. The substrate was functionalised with both p-aminothiophenol (p-ATP) Raman reporter molecules and anti-cTnI antibodies, creating a sandwich immunosensor capable of simultaneous LSPR, SERS, and thermoplasmonic readout. Following injection of plasma samples with pre-verified cTnI concentrations, antigen–antibody binding was detected as a 25 nm LSPR redshift. SERS of the same immunoreaction confirmed cTnI binding via intensity changes at 1004 and 1030 cm^−1^, corroborating the LSPR result. Thermographic imaging during laser exposure provided a third, independent confirmation through changes in the local heat profile. Whilst the overall reported clinical performance (75% sensitivity and 25% specificity) fell short of diagnostic standards and discrepancies were observed between simulated and clinical plasma performance at high cTnI concentrations, the study establishes a proof of concept for fully integrated multimodal optical–thermoplasmonic immunosensing in a microfluidic format. The independent nature of the three measurement channels provides a framework for result cross-validation that addresses a central limitation of single-mode SERS immunoassays in complex plasma [95]. 

#### 5.3.2. SERS Integrated with Electrochemistry and Acoustofluidics: Alzheimer’s Disease Biomarker Detection

A highly sophisticated multimodal diagnostic platform integrating SERS with electrochemical sensing and surface acoustic wave (SAW)-driven acoustofluidics was developed by Hao and colleagues for the detection of Alzheimer’s disease (AD) biomarkers (amyloid-β (Aβ42) peptides and tau protein) in blood plasma [100]. The platform comprised two functional components: a separation module employing SAW at tunable voltages across interdigital transducers (IDT) to achieve size-dependent fractionation of plasma constituents, and a detection module incorporating a ZnO nanorod array electrode with in situ-deposited AgNP (ZnO@Ag) for SERS, alongside a pristine ZnO electrode functionalised with biotinylated anti-Aβ42 and anti-tau antibodies for electrochemical detection.

For SERS measurements, plasma samples from HC and AD patients were introduced into the microchamber before and after acoustofluidic separation, with PCA-LDA identifying significant Raman bands at 417, 999, 1150, 1250, and 1290 cm^−1^ attributed to metabolites implicated in neurodegenerative pathology. Differences in the 600–700 cm^−1^ region between HC and AD spectra were consistent, with reduced Aβ levels in the AD group. Complementary electrochemical quantification via cyclic voltammetry and differential pulse voltammetry (DPV) established that Aβ42 concentrations in HC plasma were approximately 3.2-fold higher than in AD patients, while tau concentrations were 2.4-fold lower in HC relative to AD. The electrochemical sensing returned 90.0% sensitivity and 85.7% specificity for Aβ42 (AUC 0.97) and 71.4% sensitivity and 70.0% specificity for tau (AUC 0.84). The orthogonality of the SERS spectral fingerprinting and the targeted electrochemical immunodetection provides mutual validation of the diagnostic conclusions, while the acoustofluidic separation module reduces matrix interference without chemical pretreatment [100].

#### 5.3.3. SERS Integrated with Colourimetry and Lateral Flow: Traumatic Brain Injury Detection

For traumatic brain injury (TBI), a paper-based lateral flow strip (PLFS) was developed for the quantification of neuron-specific enolase (NSE) in blood plasma. AuNP nanostars encoded with a Raman reporter and encapsulated in a 2 nm silica shell functionalised with detection antibodies (AuNP@Reporter@SiO_2_@Ab) were adsorbed on the conjugate pad. Capture and secondary antibodies formed the test and control lines on the nitrocellulose membrane. Plasma introduced at the sample pad migrated by capillary action, and the sandwich complex formed at the test line provided both a visual grey band as a colourimetric positive control and a quantitative SERS signal at 1076 cm^−1^ used for NSE calibration. A detection range of 1.0–75.0 ng mL^−1^ with a limit of detection of 0.86 ng mL^−1^ was reported, with performance comparable to ELISA for the same samples. The dual-mode readout ensures that a positive visual result can be independently confirmed and quantified by SERS measurement, addressing the risk of false-positive colourimetric interpretation in clinical settings [172]. 

The SERS assay configurations herein highlighted, in addition to others included in Table 5, exemplify the different approaches researchers are exploring to reduce the confounding effects of variability in substrates, biological matrices and data interpretation. Several methods in Table 5 demonstrated the use of spherical NPs with/without a support matrix for small molecule detection [20,167]; anisotropic NPs [17,95]; and a variety of support matrices, Raman reporters, and sample sizes for direct and indirect SERS assays. Each process presents interesting insights, performance, and possible directions where optimisation is required and where further investigation could be impactful in improving method reliability and result reproducibility.

## 6. Data Analysis Strategies on SERS

Data analysis represents a critical step in SERS-based diagnostics, as it enables the extraction of meaningful and biochemically relevant information from complex spectral datasets [175]. In the context of plasma analysis, this task becomes particularly challenging due to the intrinsic variability of plasma composition, coupled with the heterogeneity and limited reproducibility of SERS signals [121,176]. The consequence of these factors is the generation of high-dimensional and noisy datasets, in which subtle disease-related spectral differences may become obscured [121].

**Figure 11:**
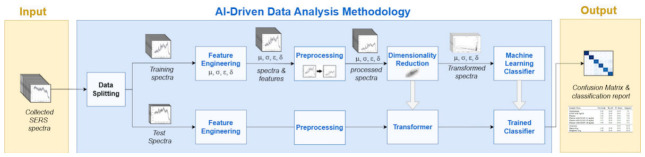
Schematic of the data preprocessing and processing methods used for SERS spectra interpretation.

Consequently, robust and well-structured computational pipelines—encompassing spectral preprocessing, feature extraction, and advanced statistical or machine learning methods—are essential not only to ensure reliable interpretation but also to enhance class separation and enable accurate and clinically meaningful classification of SERS data [18,177,178,179,180]. In Figure 11, an AI-driven data analysis methodology is represented, the various steps of the data analysis pipeline being illustrated. Collected input data is split into training and test data to enable the training of the model with a subset of the available data. The validation and evaluation of the methodology are performed using a subset of test data. The first step is the identification of key features that describe the input data. A preprocessing pipeline is then defined to improve the data quality, as described in Section 6.1. Due to the large complexity of input data, a reduction of dimensionality is then applied, as described in Section 6.2. Finally, a machine learning classifier is used as described in Section 6.3. The selection of adequate features, specific processing pipelines, dimensionality reduction mechanisms and machine learning classifiers is a highly complex data science research task with a very large number of options and possible combinations that must be validated. The state of SERS spectral interpretation is described in the following sections.

### 6.1. Spectral Preprocessing: Ensuring Data Quality for Downstream Analysis

Spectral preprocessing is a critical step in SERS data analysis, as it aims to remove artefacts, reduce noise, and standardise spectral intensity, enabling reliable comparison between samples and improving the performance of downstream analytical models [175,181,182]. Generally, SERS spectra interpretation using advanced chemometrics (AI, ML, DL, and other related algorithms) follow the sequence captured in Figure 10, progressing from data preprocessing to processing and result output. At the preprocessing stage, typical preprocessing pipelines include cosmic ray removal, smoothing or noise reduction, baseline correction to eliminate fluorescence background, and spectral normalisation to account for intensity variability [36,183] However, the effectiveness of these steps is highly dependent on both the characteristics of the dataset and the choice of analytical method. While appropriate preprocessing can enhance signal quality and facilitate pattern recognition, excessive or poorly selected processing may lead to information loss or the introduction of artefacts, resulting in a lower diagnosis accuracy [184]. Furthermore, certain deep learning approaches have demonstrated increased robustness to noise and baseline variability, allowing them to operate effectively on minimally processed spectra [184,185,186] Consequently, preprocessing strategies should be carefully optimised and validated rather than applied as a fixed protocol [181].

#### 6.1.1. Typical Preprocessing Pipelines

Cosmic ray removal, smoothing, baseline correction, and spectral normalisation constitute the typical preprocessing pipeline applied to SERS data [127,183,187]. These steps are generally performed in a sequential manner, although smoothing and baseline correction may be interchanged depending on the noise level and characteristics of the spectra [36,181,188]. Cosmic ray removal, also referred to as spike removal, is typically implemented as the first preprocessing step following data acquisition [178,187] This is due to the distinctive nature of cosmic ray artefacts, which appear as sharp, narrow peaks with abnormally high intensity and can distort spectral profiles, interfere with peak assignment, and bias multivariate statistical analyses [36,189,190]. Their well-defined spectral signature facilitates reliable detection and removal prior to the application of other preprocessing methods, which might otherwise alter their morphology and hinder accurate identification [189].

Following cosmic ray removal, smoothing and baseline correction are typically applied to further improve spectral quality [127,183,187]. The order of these steps may vary depending on the noise characteristics of the data. In cases of high noise levels, smoothing is often applied prior to baseline correction to stabilise baseline estimation and reduce the influence of high-frequency noise [36,187]. Common smoothing approaches include Savitsky–Golay filtering, which preserves peak shape while reducing noise, Whitaker smoothing, which provides robust penalised least squares-based denoising, and wavelet transform-based denoising, where spectral signals are decomposed and noise is suppressed through coefficient thresholding [17,127,191].In opposition, when the signal-to-noise ratio is relatively higher or when robust baseline algorithms are employed, baseline correction may be performed before smoothing [121]. Widely used baseline correction methods include polynomial fitting approaches, such as detrending and modified polynomial fitting, as well as more advanced techniques like asymmetric least squares (AsLS) and adaptive iteratively reweighted penalised least squares (airPLS), which incorporate regularisation strategies to effectively remove background contributions while preserving relevant spectral features [127,177,192]. Improper selection of these methods may lead to distortion of spectral features, directly impacting downstream multivariate analysis [181]. Consequently, the choice and sequence of these preprocessing steps should be adapted to the specific characteristics of the dataset, and their suitability should be determined through systematic evaluation and validation of their impact on downstream analysis.

Normalisation is a critical preprocessing step in spectral analysis, aimed at reducing intensity variability and enabling consistent comparability between spectra acquired under different experimental conditions or instrumentation [180,181]. It primarily addresses unwanted variations arising from factors such as laser power fluctuations, focus instability, and differences in sample concentration, while also improving the numerical stability of downstream data analysis [180]. This is particularly important in SERS applications, where signal variability can be exacerbated by factors such as heterogeneous enhancement and limitations in autofocus during spectral mapping.

The increasing scale of clinical SERS studies has also highlighted the need for standardised and reproducible preprocessing workflows. To address this challenge, open-source spectroscopy toolboxes such as RamanSpy [193] and PyFasma [194] have emerged as computational backbones for spectral analysis, providing unified implementations of baseline correction, normalisation, denoising, visualisation and machine learning integration. By reducing variability introduced by custom preprocessing pipelines, these frameworks facilitate reproducible analysis across research groups and clinical sites, supporting the development of large-scale multicenter diagnostic studies.

#### 6.1.2. Spectral Normalisation Strategies

A range of normalisation strategies has been developed to account for different sources of variability. Simple approaches such as min–max and maximum intensity normalisation rescale spectral intensities to a fixed range or relative scale, facilitating comparison across samples, although they remain sensitive to outlier peaks [177]. Unit vector (L2) normalisation projects each spectrum onto a unit hypersphere, ensuring uniform vector magnitude and emphasising spectral shape over absolute intensity [180]. Standard normal variate (SNV) normalisation further standardises spectra by centring and scaling each individual spectrum, effectively correcting for multiplicative scatter effects, with robust normal variate (RNV) providing an outlier-resistant alternative based on median statistics [181,195]. Area normalisation, which equalises the total spectral intensity, is particularly suitable for compensating variations in laser power or sample quantity [184]. In contrast, internal standard calibration—using a known reference molecule such as 4-mercaptopyridine (4-MP)—offers a more direct correction of SERS-specific variability by accounting for hotspot heterogeneity through a stable reference signal [121].

In the context of SERS-based diagnostics, the selection of an appropriate normalisation strategy should be guided by the dominant source of variability within the dataset. For instance, SNV or RNV are well suited for correcting scatter-related effects, area normalisation is effective in addressing power fluctuations, and internal standards provide robust correction for substrate-induced variability.

### 6.2. Dimensionality Reduction and Feature Extraction

Following spectral preprocessing, the resulting SERS spectra provide a more reliable representation of the underlying biochemical information. Nevertheless, even well-pre-processed spectra remain inherently high-dimensional, often comprising hundreds to thousands of correlated wavenumber variables, many of which may be redundant or of limited discriminative relevance [183,196]. Dimensionality reduction and feature extraction techniques are therefore applied not to compensate for poor data quality but to systematically distil the most informative spectral features and construct compact representations suitable for downstream machine learning models [180,184,197].

Without such strategies, machine learning models trained on full spectral data face two compounding challenges: an excess of variables relative to available samples, which promotes overfitting, and the strong correlation between neighbouring wavenumbers, which introduces substantial redundancy into the feature space [17,36,191]. The following subsections address the principal methods employed to overcome these challenges, progressing from dimensionality reduction through feature extraction to model interpretability.

#### Linear and Nonlinear Dimensionality Reduction

Dimensionality reduction methods transform high-dimensional spectral data into a lower-dimensional representation while preserving the most analytically relevant structure within the dataset. In SERS-based diagnostics, these methods can be broadly categorised according to whether they operate independently of class labels (unsupervised methods) or incorporate label information to guide the reduction process (supervised methods).

Principal component analysis (PCA) is the most widely used unsupervised method for dimensionality reduction in spectroscopic analysis [183,198]. It transforms high-dimensional spectral data into a set of orthogonal principal components (PCs), ordered by the variance they explain, thereby reducing dimensionality while retaining the dominant sources of variation [197]. It is commonly employed as a feature extraction step prior to supervised classification methods such as linear discriminant analysis (LDA) and Support Vector Machines (SVM) [175,179,199]. However, PCA is limited by its linear nature, which may hinder the identification of nonlinear spectral relationships, and by its sensitivity to irrelevant spectral regions, which can introduce non-informative variance into the reduced representation [121,200]. 

In addition to linear approaches, unsupervised nonlinear dimensionality reduction techniques such as t-distributed stochastic neighbour embedding (t-SNE) are increasingly used in SERS data analysis [176,201,202]. t-SNE is primarily employed as a visualisation and data quality assessment tool rather than as a feature extraction method for predictive modelling [180,201]. By projecting high-dimensional spectral data into a two- or three-dimensional embedding, it enables visual inspection of cluster structure and facilitates the identification of residual outliers not removed during preprocessing, effectively serving as a secondary validation step prior to model development [203]. However, t-SNE prioritises the preservation of local neighbourhood relationships and does not reliably maintain global inter-cluster distances, limiting its utility beyond exploratory analysis [204]. In terms of feature extraction and selection strategies, two approaches are available: algorithm-driven feature selection such as PCA loadings and PLS latent variables; and deep learning-driven extraction that includes CNN and autoencoders. Also, model interpretability and spectral attribution include VIP (Variable Importance in Projection), SHAP (Shapley Additive Explanations) and Grad-CAM (Gradient-weighted Class Activation Mapping)—this last one is usually related to CNNs. 

### 6.3. Machine Learning for Diagnostic Classification

The methodological integration of SERS with machine learning (ML) has been discussed in early reviews. Ralbovsky and Lednev [205] highlighted the potential of combining Raman spectroscopy with machine learning for medical diagnostics across a broad range of diseases, establishing its feasibility as a general diagnostic framework. In parallel, Lussier et al. [175] demonstrated that classical linear approaches are often insufficient for capturing the complexity of SERS datasets, while nonlinear machine learning models, including neural networks and deep learning architectures, provide improved capacity for spectral feature extraction and classification. While these foundational studies established the feasibility of machine learning-assisted Raman and SERS diagnostics, more recent work has focused on improving model performance and addressing persistent challenges related to data complexity, variability, and reproducibility [175].

This section focuses on machine learning approaches for SERS-based diagnostic classification, with emphasis on recent studies reported in the last few years. While foundational reviews established the feasibility of AI-driven spectroscopy up to 2020, advances in computational architectures and learning strategies have significantly expanded the field. Accordingly, this review exclusively considers high-impact literature published since 2023 to capture the current state of the art, focusing on emerging trends in SERS classification models, including deep learning architectures, resampling strategies for imbalanced datasets, and explainable AI methods, alongside persistent challenges related to dataset limitations and clinical translation.

#### 6.3.1. Classical Supervised Classifiers and Statistical Discriminants

Traditional supervised machine learning algorithms and statistical discriminants form a foundational benchmark across the reviewed cohort due to their efficiency when applied to tabularized spectral features. Within this framework, models are primarily used to establish robust, hyper-dimensional decision boundaries for binary and multi-class clinical diagnostic partitions.

##### Support Vector Machines

Support Vector Machines (SVMs) are supervised learning algorithms that construct optimal separating hyperplanes by maximising the margin between classes defined by support vectors [206]. In SERS-based diagnostic classification, nonlinear separability is commonly addressed through kernel functions, particularly Gaussian or radial basis function (RBF) kernels, which enable classification in higher-dimensional feature spaces [207,208,209]. The suitability of SVMs for SERS analysis is largely associated with their strong generalisation performance in high-dimensional datasets and robustness under limited sample sizes [207,208,209]. Across the recent literature cohort, SVMs remain among the most widely applied classifiers for SERS-based diagnostics. Recent studies have employed SVM models for sepsis stage stratification [210], rapid detection of drugs in blood under complex circulatory backgrounds [211], detection of large-scale pan-cancer panels using massive validation cohorts [212], and differentiation of chronic autoimmune disorders from healthy matrices [49,213].

##### Linear and Quadratic Discriminant Analysis (LDA/QDA)

Linear discriminant analysis (LDA) and Quadratic Discriminant Analysis (QDA) are supervised classification methods that construct decision boundaries to separate classes in multivariate datasets. LDA maximises the ratio of between-class variance to within-class variance under the assumption that all classes share the same covariance matrix, resulting in linear decision boundaries [214]. QDA relaxes this assumption by allowing class-specific covariance structures, producing quadratic decision boundaries that can better model heterogeneous class distributions [215]. Because spectral datasets typically contain substantially more variables than samples, LDA and QDA are commonly combined with dimensionality reduction techniques such as PCA, yielding workflows such as PCA-LDA and PCA-QDA [205].

In SERS-based diagnostics, LDA/QDA approaches remain widely used due to their interpretability, computational efficiency, and suitability for moderate-sized spectral datasets, including the classification of allograft kidney transplant rejection [216], identification of urinary disease biomarkers [217], profiling of circulating plasma cell malignancies [218], characterisation of plasma extracellular vesicles [219], and serum fingerprint analysis for disease discrimination [49,220]. Although LDA-based models remain effective baseline classifiers in SERS diagnostics, their performance may become limited in highly heterogeneous datasets or under complex nonlinear class distributions, where more flexible machine learning approaches can provide improved discrimination performance [205]. 

##### Partial Least Squares Discriminant Analysis (PLS-DA)

Partial Least Squares Discriminant Analysis (PLS-DA) is a supervised multivariate classification method that combines partial least squares regression with discriminant analysis to separate predefined sample classes based on high-dimensional feature sets [221,222]. In PLS-DA, latent variables are extracted to maximise both the variance explained in the predictor matrix and the covariance between predictor variables and class membership labels. Due to its ability to handle highly collinear and high-dimensional datasets, PLS-DA is widely applied in spectroscopic and chemometric analysis of SERS data [221,223].

In SERS-based diagnostics, PLS-DA is particularly suitable for analysing complex spectral profiles containing large numbers of correlated variables, where conventional linear classifiers may become unstable [221,223]. The method also provides biochemical interpretability through latent-variable loadings and variable importance metrics, enabling identification of spectral regions contributing to class discrimination [222,224]. Because SERS datasets frequently contain substantial spectral variability and multicollinearity, PLS-DA and related variants are commonly used following spectral preprocessing and dimensionality reduction workflows [223].

More recent studies have continued to apply PLS-DA-based approaches for the classification of complex SERS diagnostic datasets. PLS-DA and PC-PLS-DA variants have been employed for tracking multiplexed exosome populations [225] and identifying respiratory disease markers from SERS spectral profiles. These studies further support the utility of PLS-DA as a robust regression-discriminant framework for the classification of multicomponent SERS spectra with inherent collinearity. Despite its effectiveness, PLS-DA remains susceptible to overfitting in high-dimensional datasets with limited sample sizes, requiring rigorous validation strategies and careful handling of nested spectral structures during model development [221,223].

#### 6.3.2. Tree-Based Ensemble Frameworks and Automated ML (AutoML)

Tree-based ensemble methods constitute an important class of supervised machine learning approaches in SERS-based diagnostics due to their ability to model nonlinear relationships in high-dimensional spectral datasets [205,226]. These methods combine multiple decision trees to improve predictive robustness, reduce overfitting through ensemble averaging, and provide variable importance measures that facilitate interpretation of diagnostically relevant spectral regions [26]. Among the most commonly used frameworks are Random Forests (RF), gradient boosting architectures, and more recent Automated Machine Learning (AutoML) systems.

Random Forests (RF) are ensemble models constructed from multiple decision trees generated using bootstrap sampling and randomised feature selection during node splitting [26]. Their ability to handle high-dimensional spectral variables without extensive feature engineering has made them suitable for SERS analysis [26]. Recent studies have applied RF-based approaches for the identification of colon malignancies [227] and the optimisation of complex internal calibration models for SERS quantification workflows [228]. Gradient boosting methods iteratively construct ensembles of weak learners, where each subsequent tree minimises the errors of preceding models [26]. Among these approaches, eXtreme Gradient Boosting (XGBoost) has gained prominence due to its computational efficiency and strong predictive performance [229,230]. Recent applications in SERS diagnostics have employed XGBoost architectures to develop highly accurate classification frameworks across broad concentration ranges in targeted immunoassays [231].

More recently, Automated Machine Learning (AutoML) frameworks have emerged as a strategy to automate model selection, hyperparameter optimisation, and ensemble construction within SERS diagnostic pipelines [15]. AutoML systems such as AutoGluon integrate multiple machine learning architectures, including Random Forests, boosting frameworks and regularised regression models into stacked ensemble structures optimised automatically for a given dataset. Recent studies have applied AutoGluon-based frameworks for automated clinical staging and cancer subtype classification from SERS spectra [232], illustrating the growing transition from manually optimised pipelines toward fully automated ensemble-learning strategies in SERS-based diagnostics.

Despite their strong predictive performance, tree-based ensemble frameworks and AutoML approaches generally require careful validation to avoid overfitting and to ensure model generalizability across heterogeneous spectral datasets [205,226].

#### 6.3.3. Deep Neural Networks and Advanced Convolutional Topologies

Deep neural networks (DNNs) are computational architectures composed of multiple interconnected layers capable of learning hierarchical feature representations directly from raw input data [233,234]. In SERS-based diagnostics, deep learning approaches have gained prominence due to their ability to automatically extract discriminative spectral features without extensive manual feature engineering [233,235]. Among these methods, convolutional neural networks (CNNs) represent the most widely adopted architectures for spectral classification tasks. CNNs employ convolutional kernels to identify local spectral patterns through sequential convolutional, pooling, and fully connected layers [234,235]. More advanced convolutional topologies, including Residual Networks (ResNet), Fully Convolutional Networks (FCN), and multiscale convolutional architectures, have further extended the capacity of deep learning models to analyse complex spectral datasets [233].

One-dimensional convolutional neural networks (1D-CNNs) remain central to recent SERS classification workflows, operating directly on spectral vectors to capture local vibrational features. Recent studies have implemented custom 1D-CNN architectures composed of sequential convolutional blocks incorporating Batch Normalization, ReLU or LeakyReLU activation layers, max-pooling operations, and fully connected layers with Dropout Regularisation to improve classification robustness [236]. More recently, two-dimensional convolutional strategies have emerged through structural conversion of spectral vectors into 2D matrix representations prior to CNN processing. These 2D-CNN pipelines employ multi-filter convolutional architectures and SoftMax-based classification layers for clinical discrimination tasks. Such approaches extend earlier demonstrations that matrix restructuring of spectral data can improve CNN classification performance by enabling extraction of spatially correlated spectral features [33].

Recent deep learning developments have produced architectures specifically designed for spectroscopic data rather than adapted from image-analysis frameworks. Examples include Spectra Segmentation Networks (SSNet) [237] and Raman Analysis through Deep Adaptive Representation (RADAR), which are designed to accommodate the one-dimensional structure of spectral signals while performing automatic feature extraction without manual peak selection [238]. Unlike traditional machine learning pipelines that depend on handcrafted spectral features, these end-to-end architectures learn hierarchical representations directly from raw or minimally processed spectra. By simultaneously identifying diagnostically relevant spectral regions and performing classification, they reduce analyst bias and improve scalability for large clinical datasets.

Advanced residual and pre-trained neural architectures have also become increasingly prominent in SERS diagnostics. Residual Networks (ResNet and ResNet18), originally developed to mitigate vanishing-gradient effects in deep architectures through skip connections [187], have recently been adapted for tracing exosome signatures and thyroid cancer staging from SERS spectra [239,240]. In parallel, pre-trained visual recognition backbones, including AlexNet, VGGNet, GoogLeNet, SqueezeNet, and DenseNet, together with recurrent neural networks (RNNs) and temporal convolutional networks (TCNs), have been integrated into custom multiscale fusion convolutional neural networks (MCNNs) to capture localised vibrational variations in spectral data [177]. Beyond convolutional approaches, recent studies have also implemented deep multilayer perceptron (MLP) architectures for ultrasensitive blood-based target tracking from SERS measurements [241]. These frameworks employ optimisation strategies such as Rectified Adam (RAdam) and weighted cross-entropy loss functions to stabilise gradient updates during training.

Compared with traditional machine learning methods, deep learning frameworks offer end-to-end learning capabilities, scalability to increasingly large spectral datasets, and improved performance in complex multi-class classification settings [187,233,234,235]. However, these architectures generally require substantially larger annotated datasets and careful validation to ensure generalisation across heterogeneous SERS platforms and clinical cohorts [235].

#### 6.3.4. Resampling Architectures and Data Augmentation

A major challenge in translating surface-enhanced Raman spectroscopy (SERS) into clinically robust diagnostic systems is the intrinsic imbalance and limited size of biomedical spectral datasets, where large healthy cohorts are frequently contrasted against comparatively sparse pathological populations [214,242]. These asymmetries can substantially bias machine learning classifiers, particularly in high-dimensional spectral settings, motivating the development of dedicated resampling architectures, synthetic sample generation strategies, and hybrid augmentation frameworks [243].

Synthetic oversampling methods are among the most widely adopted approaches for mitigating class imbalance in spectroscopic datasets. The Synthetic Minority Oversampling Technique (SMOTE) generates artificial minority-class samples through interpolation between neighbouring minority observations, thereby balancing the class distribution without direct duplication [244]. Variants such as ADASYN (Adaptive Synthetic Sampling) further refine this concept by adaptively generating more synthetic samples in regions where minority examples are harder to learn, effectively shifting the decision boundary toward difficult cases [244]. More recent biomedical SERS studies also report the use of BorderlineSMOTE to preferentially generate synthetic samples near class boundaries, where diagnostic ambiguity is greatest.

To address the introduction of noisy or overlapping synthetic samples, hybrid resampling–cleaning strategies have become increasingly important. Methods such as SMOTEENN combine SMOTE with Edited Nearest Neighbours (ENN), removing samples whose neighbourhood structure suggests class inconsistency, whereas SMOTETomek integrates SMOTE with Tomek link removal to eliminate overlapping majority–minority sample pairs [245,246]. Recent studies have further integrated these hybrid resampling architectures with outlier-detection frameworks such as PCA-DBSCAN to improve algorithmic fairness and reduce minority-class omission during large-scale pan-cancer screening workflows [247]. Beyond classical oversampling, data augmentation techniques specifically designed for Raman and SERS spectra have become increasingly relevant for deep learning applications. Di Frischia et al., [248] proposed a hierarchical augmentation pipeline incorporating additive Gaussian noise, baseline correction variants, smoothing filters, clustering-based balancing, spectral shifting, and Generative Adversarial Networks (GANs) for synthetic Raman spectrum generation. CNN classifiers trained on GAN-augmented datasets achieved improved classification performance relative to non-augmented training schemes [248]. Similarly, Weng et al. [233] demonstrated that reshaping one-dimensional SERS spectra into two-dimensional matrices substantially improved CNN performance for both classification and quantitative prediction tasks, while simple spectral intensity perturbations also enhanced model robustness [233].

These augmentation and resampling approaches are particularly important in SERS-based medical diagnostics, where spectral heterogeneity, hotspot variability, and limited patient availability can compromise generalisation performance. Contemporary studies therefore increasingly rely on integrated resampling pipelines combining synthetic generation, boundary cleaning, and deep learning-oriented augmentation to stabilise classification models and improve minority-class sensitivity in clinically imbalanced datasets. Beyond conventional regularisation approaches, specialised spectroscopy-oriented architectures have recently been proposed to preserve the physical interpretability of Raman spectra during model training. Peak-Sensitive Elastic-Net Regularisation (PSE-LR) extends traditional Elastic-Net methods by assigning greater importance to diagnostically relevant Raman bands while penalising model dependence on spectral background fluctuations [249]. By encouraging feature selection around known biochemical signatures, PSE-LR helps maintain the physical integrity of spectral interpretations and reduces the risk of models exploiting noise-driven correlations. Such approaches represent an important step toward bridging predictive performance and explainability in clinical spectroscopy.

#### 6.3.5. Evaluation Metrics, Cross-Validation, and Generalisability

The performance of surface-enhanced Raman spectroscopy (SERS)-based diagnostic models must be rigorously evaluated to ensure reproducibility, robustness, and clinical applicability. In SERS studies, classification performance is commonly assessed through confusion-matrix-derived metrics, including accuracy, sensitivity, specificity, precision, and F1-score [250]. Sensitivity measures the ability of a model to correctly identify diseased samples, whereas specificity quantifies the correct identification of healthy samples [250]. Because SERS datasets are frequently imbalanced, balanced accuracy is often preferred over overall accuracy, as it equally weighs performance across classes [251]. Precision and F1-score further evaluate the trade-off between false positives and false negatives, particularly in uneven class distributions [250].

Receiver Operating Characteristic (ROC) curves and the corresponding area under the curve (AUC) are also widely employed to evaluate discriminative capability across multiple classification thresholds [205,251]. These metrics are particularly important in diagnostic applications because they quantify the trade-off between sensitivity and specificity and allow comparison between different classification models [250]. Recent SERS studies continue to employ standardised metrological evaluation schemes. Contemporary diagnostic models are commonly assessed using overall accuracy, balanced accuracy, sensitivity (recall), specificity, precision, F1-score, and classification success rate (CSR) in both targeted assays and broad screening applications [252,253]. In addition, ROC curves and AUC calculations are consistently reported to quantify true-positive versus false-positive trade-offs across varying decision thresholds.

Robust validation strategies are essential to prevent overfitting and to ensure that SERS classification models generalise to unseen data [223,254]. Common approaches include k-fold cross-validation, Leave-One-Out Cross-Validation (LOOCV), and Repeated Double Cross-Validation (RDCV) [251,255]. 

In k-fold cross-validation, the dataset is partitioned into k subsets, with iterative training and testing performed across folds [251,255]. RDCV extends this concept through nested validation loops that separate model optimisation from performance assessment, thereby reducing the risk of information leakage and overestimation [251]. Guo et al., [254] highlighted critical methodological pitfalls in spectroscopic model validation. First, splitting datasets at the spectrum level rather than at the biological replicate or patient level can produce artificially inflated accuracies because spectra from the same sample are not independent. Second, dimensionality reduction methods such as PCA or PLS must be performed inside the cross-validation loop to avoid leakage of information from validation data into the model [254]. These recommendations are particularly relevant for SERS datasets, which often contain hierarchical replicate structures and high-dimensional spectral variables [223,254].

Recent studies increasingly avoid simple train/test splits and instead implement more rigorous validation protocols to ensure generalisability. Current approaches predominantly employ 5-fold cross-validation schemes or independent geographically isolated clinical verification cohorts for external validation. Such strategies improve the reliability of reported diagnostic performance and better reflect real-world deployment conditions. Additional validation procedures such as permutation testing are also used to assess whether classification performance exceeds chance expectations [255]. By repeatedly randomising class labels and rebuilding models, permutation testing generates null distributions for performance metrics, allowing statistical significance to be quantified [255]. Because SERS spectra are highly sensitive to substrate variability, hotspot distribution, environmental conditions, and preprocessing choices [223,254], rigorous validation and transparent reporting remain fundamental requirements for clinically reliable SERS-based diagnostic systems.

#### 6.3.6. Explainable AI (XAI) and Model Interpretability Modalities

Deep learning models applied to surface-enhanced Raman spectroscopy (SERS) achieve strong diagnostic performance but are often described as “black-box” systems, where the internal reasoning behind predictions is not directly interpretable [175]. Explainable AI (XAI) addresses this limitation by providing methods that reveal how models reach their decisions, improving transparency, trust, and clinical reliability. In SERS-based diagnosis, these methods are particularly important because they allow model outputs to be linked back to physically meaningful spectral signatures. Explainable AI bridges the gap between deep learning predictions and the physical basis of SERS by transforming abstract model activations into interpretable attribution maps associated with specific spectral regions and wavenumbers [232,233]. These explanations allow researchers and clinicians to determine whether model predictions are driven by diagnostically relevant vibrational signatures rather than artefacts arising from instrumental variability, background interference, or noise [222,223]. As a result, XAI provides a mechanism for validating that learned representations remain consistent with established spectroscopic principles and biochemical knowledge.

##### Gradient-Based Visualisation Methods

Contemporary SERS diagnostic frameworks routinely incorporate gradient-based interpretability techniques, including Class Activation Mapping (CAM), Grad-CAM (Gradient-weighted Class Activation Mapping), and Integrated Gradients (IG). These approaches generate contribution maps that identify which input regions or spectral features are most influential for a given prediction [18,247]. 

From a foundational perspective, CAM produces class-specific activation maps by using feature representations from the final convolutional layers of a CNN [256]. However, CAM heatmaps are often low-resolution due to the coarse spatial structure of deep feature maps. Grad-CAM extends this idea by using gradients of the target class with respect to feature maps, enabling class-discriminative localisation without modifying the network architecture [257]. Integrated Gradients provides a complementary attribution mechanism by computing the integral of gradients along a path from a baseline input to the actual sample, ensuring theoretically grounded feature attribution [258].

In SERS applications, these gradient-based methods are used to generate heatmaps over spectral inputs, allowing identification of specific wavenumbers that drive diagnostic decisions. This enables direct mapping between model behaviour and molecular vibrational information [175,233]. By linking model predictions to specific spectral features, XAI enables comparison between algorithmic decision-making and established vibrational spectroscopy assignments [18,187,253]. For example, in studies targeting neurodegenerative diseases, attribution maps can identify whether predictions are associated with diagnostically relevant bands such as the 1607 cm^−1^ uric acid/tryptophan feature or the 1157 cm^−1^ NeuAc-related vibration [18]. This correspondence provides a physically interpretable rationale for model outputs and strengthens confidence that classification decisions are based on meaningful biochemical information rather than spurious correlations.

##### Game-Theoretic Feature Attribution (SHAP)

In addition to gradient-based approaches, modern SERS diagnostic pipelines employ game-theoretic interpretability methods, particularly SHAP (Shapley Additive Explanations). SHAP assigns additive feature contributions based on Shapley values from cooperative game theory, distributing the model prediction among input features according to their marginal contribution [259,260]. In ensemble and tree-based frameworks, SHAP provides a principled decomposition of predictions into feature-level contributions, enabling clinicians to verify which spectral variables most strongly influence diagnostic outcomes [232]. This makes SHAP particularly valuable for interpreting SERS data, where each feature corresponds to specific wavenumber regions potentially associated with biochemical signatures. By employing game-theoretic SHAP values, each diagnostic prediction can be decomposed into contributions from individual spectral features. This enables quantification of how specific wavenumber regions influence the probability assigned to a disease class, providing a transparent connection between model outputs and the molecular vibrations represented within the spectrum [232]. Consequently, SHAP-based explanations help ensure that predictive performance can be interpreted in the context of underlying biochemical and spectroscopic mechanisms.

##### Interpretability in SERS-Based Diagnostics

SERS combined with machine learning has become a powerful diagnostic platform due to its ability to generate molecular fingerprint spectra with high sensitivity for disease detection [170,231]. However, the high dimensionality and complexity of spectral data require interpretable machine learning techniques to ensure that predictions are clinically meaningful.

Recent SERS studies show that interpretability methods are increasingly integrated into diagnostic pipelines to ensure that model decisions reflect genuine spectral biomarkers rather than spurious correlations. Gradient-based attribution methods highlight discriminative spectral regions, while SHAP-based explanations quantify feature contributions in a model-agnostic and clinically interpretable manner [232]. Together, these methods provide complementary insights: gradient-based methods offer local sensitivity maps over spectral inputs, whereas SHAP provides global and local feature importance grounded in additive attribution theory.

To provide a comprehensive and structured overview of how these diverse computational approaches map onto the current state-of-the-art landscape, Table 6 categorises the reviewed contemporary literature (2023–present). This matrix groups studies according to their principal algorithmic families, highlighting the corresponding model architectures, evaluation methodologies, and the range of performance outcomes reported across a variety of clinical diagnostic applications.

For example, Kim et al. [18] employed XAI methods to quantify the contribution of individual spectral features to model predictions, enabling direct association between diagnostic performance and specific metabolite-related Raman bands. Such approaches strengthen the interpretability of SERS-based classifiers by linking predictive outcomes to chemically meaningful spectral signatures rather than relying solely on aggregate performance metrics.

### 6.4. Data Landscape in Spectroscopic AI

The performance, reproducibility, and generalisability of machine learning and deep learning models in SERS-based diagnostics are fundamentally constrained by the volume, structure, and validation strategy of the underlying datasets. To systematise the heterogeneous 2023–present literature, reviewed studies are summarised according to three key dimensions: (i) data composition and biological matrix, (ii) cohort scale and sampling strategy, and (iii) validation and data-splitting protocols. This structured mapping highlights how differences in experimental design directly influence reported model performance and generalisation claims.

Across these categories, a consistent pattern emerges: early-stage SERS studies tend to rely on small exploratory cohorts combined with aggressive spectral inflation strategies, whereas more recent studies increasingly adopt larger multi-cohort datasets and stricter validation protocols such as k-fold cross-validation and external cohort testing. This shift reflects a broader transition in the field from proof-of-concept spectral classification toward clinically deployable diagnostic systems, where generalisability across instruments, populations, and acquisition conditions becomes a central requirement. Importantly, the distinction between *patient-level data* and *spectrum-level data* remains a critical confounding factor. Many studies inflate limited patient cohorts into large spectral datasets, which can artificially improve reported performance unless validation is performed at the correct hierarchical level. As summarised in Table 7, only studies employing external or allopatric validation strategies provide strong evidence of true cross-population robustness.

A critical distinction must be maintained between spectrum-level splitting and patient-level splitting. In SERS-based artificial intelligence workflows, dataset integrity is compromised when multiple SERS spectra acquired from the same patient sample are treated as independent observations. If these non-independent spectra are distributed across both training and testing folds, severe data leakage occurs, allowing the model to learn patient-specific characteristics, substrate-related artefacts, or acquisition-specific signatures rather than disease-associated biochemical features. As a result, reported performance metrics may be artificially inflated and fail to reflect true generalisation to unseen patients.

To mitigate this issue, data partitioning should be performed at the patient level, ensuring that all SERS spectra originating from a given individual are assigned exclusively to either the training, validation, or testing set. Patient-level splitting is therefore considered a prerequisite for reliable model evaluation and should be complemented, whenever possible, by external cohort validation to assess robustness across different patient populations, acquisition conditions, and SERS platforms.

Nevertheless, practical limitations such as rare diseases, limited sample availability, and pilot-scale studies often result in small patient cohorts. In these situations, multiple SERS spectra acquired from the same patient may still provide valuable information for model development and data augmentation. However, augmentation procedures should only be applied after patient-level partitioning has been performed, with all original and augmented spectra remaining within the same dataset subset. Strategies such as intensity scaling, spectral perturbation, baseline variation, noise injection, and synthetic spectrum generation can improve model robustness and training stability, but they cannot replace the biological diversity obtained from independent patient cohorts. Consequently, augmentation should be viewed as a complement to, rather than a substitute for, rigorous patient-level validation.

### 6.5. Computational Challenges and Methodological Bottlenecks

Despite the high performance reported in recent SERS-based machine learning studies, clinical translation remains limited by several computational and methodological constraints. These challenges are primarily driven by the high-dimensional nature of spectral data, inconsistencies in sampling design, and the lack of standardised validation and interpretability frameworks.

#### 6.5.1. Dimensionality and Complexity Constraints

SERS datasets are inherently high-dimensional, containing thousands of wavenumber variables per spectrum. While these features encode rich biochemical information, disease-relevant variations are often subtle and not directly observable, requiring complex feature extraction pipelines [212]. Dimensionality reduction methods such as PCA are widely used for computational efficiency; however, excessive reliance on linear projections may oversimplify nonlinear spectral relationships and reduce classification performance in heterogeneous clinical data [263]. This introduces a fundamental trade-off between efficiency, interpretability, and information preservation.

#### 6.5.2. Overfitting and Pseudoreplication

A major limitation in current studies is the mismatch between the number of spectra and the number of independent patients. Multiple spectra acquired from the same subject are often treated as independent samples, leading to pseudoreplication. As a result, models may learn patient-specific or acquisition-related artefacts rather than disease-relevant signals [213,236], inflating reported performance and limiting generalisability.

Data augmentation and synthetic sampling can mitigate class imbalance and improve training stability, but they may also blur class boundaries if not properly constrained, potentially reducing sensitivity in real clinical conditions.

#### 6.5.3. Spectral Instability and Concentration Effects

SERS signals are highly sensitive to sample preparation conditions, particularly dilution and concentration. Insufficient or excessive dilution can respectively introduce substrate heterogeneity or reduce analyte availability, leading to unstable spectral inputs [263]. At high concentrations, signal saturation further limits quantitative reliability due to nonlinear intensity responses [211]. These effects collectively reduce reproducibility across experimental and clinical settings. Beyond concentration-dependent variability, SERS measurements are inherently affected by noise arising from stochastic hotspot formation, nanoparticle aggregation, fluorescence background, and local substrate heterogeneity. Consequently, recent research has increasingly focused on improving signal-to-noise ratio (SNR) and measurement reproducibility rather than solely maximising enhancement factors. Several complementary approaches have emerged to address these limitations. At the computational level, spread-spectrum SERS (SS-SERS) distributes spectral information across a broader frequency domain and reconstructs signals through correlation-based decoding, enabling substantial suppression of uncorrelated noise and fluorescence background while achieving attomolar detection of neurotransmitters [264]. At the substrate level, hybrid plasmonic platforms incorporating two-dimensional materials such as graphene, MoS_2_, and WSe_2_ have demonstrated reduced spectral variability, fluorescence quenching, and significant SNR improvements compared with conventional metallic nanoparticle substrates [265]. Noise reduction can also be achieved through optimisation of the optical acquisition system. For example, rough-cutting the end surface of fibre-optic SERS probes has been shown to reduce optical artefacts and improve signal collection efficiency, resulting in measurable SNR gains [266]. Collectively, these developments reflect a broader transition from enhancement-driven substrate design towards integrated strategies that simultaneously optimise sensitivity, reproducibility, and noise suppression, which are increasingly recognised as essential requirements for clinical translation of SERS-based diagnostic systems.

#### 6.5.4. Biomolecular Heterogeneity and Class Overlap

Systemic biofluids exhibit strong biological variability, including inflammation, comorbidities, and inter-individual differences, which contribute to overlapping spectral signatures [263]. This is particularly problematic for early-stage disease detection, where weak biomarker signals are easily masked by physiological noise. Consequently, while binary classification tasks involving advanced disease often achieve high accuracy, performance typically degrades in multi-class or early diagnostic scenarios [212,262].

#### 6.5.5. Explainability and the Clinical Adoption Barrier

Although deep learning models such as CNNs and ensemble architectures achieve strong predictive performance, their “black-box” nature remains a major barrier to clinical adoption [232]. Clinical decision-making requires transparent justification of predictions, which motivates the integration of explainable AI methods such as SHAP, Grad-CAM, and Integrated Gradients. However, these approaches introduce additional computational overhead and lack unified clinical validation standards, limiting their current regulatory acceptance [18,247,261]. 

## 7. Future Perspectives

This final section highlights the hurdles preventing the translation of SERS from the research laboratory to clinical application. Challenges are categorised according to substrate, biological matrix, data interpretation, and experimental/equipment setting protocols. A summary of opportunities within the SERS landscape is also presented, with a few recommendations.

### 7.1. Challenges

There are exhaustive reports detailing key challenges that must be addressed before SERS can become accepted as a clinical diagnostic technique [267]. Research papers reporting extremely low limits of detection (LOD) and high clinical sensitivity and selectivity for different disease biomarkers support the utility of SERS in clinical diagnosis. However, there is also a widely acknowledged limitation regarding poor method reliability and result reproducibility [267,268]. Among the underpinning causes is that SERS is dependent on surface chemistry and adsorption dynamics, whose mechanisms are yet to be fully elucidated. So, while there are optimistic reports on figures of merit, there is sparse information detailing the exact experimental conditions that make most of the outstanding results possible. Neither are in-depth scientific explanations available that increase our understanding of analyte–substrate interactions, their control mechanisms, and their means of consistent reproduction [268,269]. Based on the general workflow of SERS-based assays, several factors contribute to variability and reproducibility challenges, including substrate fabrication and properties, sample matrix complexity, data processing methods, experimental protocols, and spectrometer calibration. Together, these factors underlie the intra- and interlaboratory variations commonly observed in SERS analyses [9,270].

#### 7.1.1. Substrate-Base Challenges

In terms of substrates for quantitative measurements, the diversity of substrate fabrication methods available, with their respective advantages and disadvantages, means there is no set standard in the choice of substrate or fabrication methods. This diversity in fabrication methods results in substrates with diverse physicochemical surface characteristics, leading to differences in SERS efficiency, stability, and repeatability [162]. The EF from analyte molecules within hotspots and outside hotspots vary by many orders of magnitude. Similarly, slight variations in substrate geometry result in variation in field enhancement [9]. Although advanced fabrication methods such as lithography produce highly reproducible substrate arrays, hotspots disparity per substrate and batch persists [36,37].

For colloidal substrates, an additional complexity arises whereby the storage conditions and age of colloids prepared by similar methods can lead to different surface affinities for a given identical species, causing fluctuations in measurements [268]. Notably, surface rearrangement events at transient and longer timescales are induced by light, thermal, or plasmonic electron transfer events, which create fluctuations in single-molecule and high-speed acquisition experiments. Additionally, during measurements, temperature change due to the interaction with lasers might result in deformation and rearrangement of the original NP substrate surface. It could also cause thinning or clustering of the adsorbed target analyte molecules on the substrate surface, impacting the measurements and spectra generated [268]. All these factors, impact in inter-batch variation and, consequently, the reproducibility and reliability of SERS measurements. A key strategy proposed to reduce variability arising from substrate fabrication is comprehensive post-synthesis characterisation, including assessment of substrate sensitivity, uniformity, reproducibility, and long-term stability. Achieving robust standard operating procedures (SOPs) for these parameters will likely require coordinated interlaboratory collaboration [267].

#### 7.1.2. Biological Matrix Challenges

Another source of variation in SERS measurement reproducibility is the biological matrix. For example, plasma is a complex matrix with thousands of proteins, nucleic acids, metabolites and other compounds. On average, the proportion of plasma (and other biofluids) constituents is normally stable, but plasma heterogeneity arises during some pathologic conditions unique to each individual [173]. Moreover, plasma is dominated by high molecular weight protein fractions (HMWF) in high abundance, with several distinct domains whose interaction with NP surfaces results in poor reproducibility [36]. In addition to this, the aggregation observed in label-free SERS when suspensions of SERS substrates are used is attributed to the high electrolyte content in plasma, which shields the stabilising surface charge on the NP substrate, leading to aggregation. HMWF readily adsorb on the NP surface, forming a “protein corona”, with protein-NP binding equilibria influencing spectral profiles [9,162,271]. Also, variation in the quality (degradation) of the biological matrix impacts the measured spectra. In addition, constituents such as uric acid, hypoxanthine and carotenoids, which have a higher affinity for NP surfaces, outcompete target analytes for hotspots, thus generating intense signals that dominate and possibly confound SERS spectra [161,162,163]. Notably, the flux present when molecules within the plasma sample diffuse in and out of the hotspot also adds to signal variation [9]. Undoubtedly, SERS can generate outstanding qualitative and quantitative information about target analytes present in complex matrices such as blood plasma, yet the impact of pre-analytical bias arising from sample size, genetic variation, race, sex, and other factors has not been evaluated comprehensively, hence the difficulty in generalising many reported clinical metrics [132]. This is why many papers emphasise further validation using a larger sample size.

#### 7.1.3. Data Analysis Challenge

SERS spectra generated from plasma and other biological matrices are affected by high dimensionality, baseline fluorescence, cosmic ray spikes, and matrix-induced noise, which requires cleaning up to remove artefacts that might confound and invalidate results from the final data processing [123]. Traditional and advanced statistical analytical methods are mandatory to convert complex SERS spectra data into clinically relevant information [272]. Notably, unsupervised learning algorithms (e.g., Principal Component Analysis, PCA; Hierarchical Cluster Analysis, HCA) enable exploratory pattern recognition without prior class labels. Supervised learning methods (e.g., Partial Least Squares Discriminant Analysis, PLS-DA and Support Vector Machines, SVMs), by contrast, exploit known class information for predictive modelling. Artificial intelligence (AI) algorithms (convolutional neural networks, CNN; machine learning, ML; and deep learning, DL) extend these capabilities through end-to-end learning. The most relevant of these statistical strategies have been detailed in Section 6.

However, as with substrate fabrication, the wide variety of available propriety data analysis software; in-house and publicly accessible statistical platforms; and several simple and advance algorithm models employed in SERS spectral preprocessing, processing and postprocessing all contribute to reproducibility challenges in SERS analyses. From preprocessing comprising of spike removal, baseline correction, smoothing and related data pretreatment, the choice of data analysis method is arbitrary and subjective, as no statistical protocol exists that is generally accepted as standard [36,123]. This unavailability of a central data processing standard is evident from the variety of chemometric methods utilised in several studies highlighted in Section 5 and discussed in Section 6.

#### 7.1.4. Experimental and Equipment Challenges

The general experimental protocols/parameters and spectrometer settings used also contribute to the reliability and reproducibility challenge. As with substrate fabrication or data analysis, there is no standard experimental protocol available; therefore, there is limited information that details how the SERS spectra are affected by sample variability and pretreatment methods; the selected experimental parameters with respect to equipment and substrate; and the optimal biological matrix for a particular SERS assay [162]. The diversity of manufactured spectroscopic equipment also introduces a level of variation to SERS measurements due to component and parameter settings. Several interlaboratory studies from different research groups to evaluate and standardise experimental and measurement parameters acknowledge the difficulty in overcoming extant issues regarding reliability and reproducibility [267,273,274,275]. A typical example is that compared to conventional techniques like ELISA with validated reproducibility ≥15% [56], the most successful parameters tested for select substrates and laser excitation wavelengths in interlaboratory studies produced an average square error of prediction (SEP) that reached 12%, which although was low, yet did not meet the criteria for a quantitative measurement (1/SEP > 15) [267]. Among identified causes, were differences in the Raman equipment brands and manufacturer-specific setups [273]. Another challenge is the polarisation sensitivity of plasmonic nanoparticles, as SERS enhancement depends not only on nanoparticle geometry but also on the incident polarisation and structured-light excitation used. Variations in near-field distribution and Raman tensor coupling can significantly affect signal intensity and spectral response, complicating the standardisation of AI-assisted plasma diagnostic platforms [276].

#### 7.1.5. Opportunities

The challenges outlined above are not impossible to resolve. Across substrates, biological matrices, data analysis, and experimental infrastructure, the SERS research community has identified the sources of SERS variability and demonstrated, in the reported studies, the diverse approaches capable of mitigating each of the challenges. Hence, several approaches are now routinely incorporated in SERS assays to improve reproducibility at the substrate, biological matrix, data interpretation, experimental parameter, and equipment setting measurement levels [36]. For the substrate, irrespective of the fabrication method chosen, a detailed post-synthesis characterisation is imperative to evaluate substrate sensitivity, uniformity, reproducibility, and longevity, with interlaboratory collaboration encouraged as a means of developing an optimal standard operating procedure (SOP) [267]. Current advances in substrate design and fabrication have helped reduce issues with batch-to-batch reproducibility [56]. There are now technological tools that can fabricate NPs with precise geometries and interparticle gaps, as well as prepare colloidal/solid substrates and nanotags with high fidelity, primed for single and multiplex detection. While advanced technology has not completely solved the reproducibility issues, it has supported the drive towards standardisation [36,56].

For variations arising from the biological matrix, adhering to good laboratory practices (GLP) could mitigate aspects of pre-analytical variations during sample collection and storage. Sample pretreatment, namely filtration, serum fractionation and protease inhibitor treatment, is encouraged to reduce the impact of protein corona and band masking whilst avoiding the degradation of important proteins, such as biomarkers. Reports suggest plasma/serum protein filtration does not result in loss of SERS spectral information [39,123,162]. It is evident that most SERS-based studies on disease diagnostics have progressed to analysing limited clinical samples, with high predictive accuracy identifying healthy and unhealthy samples. However, clinical testing on a larger sample population is required to validate reported experimental results, an important step toward approval for general diagnostics [56].

Apart from the challenges pertaining to measurement variability, the development of affordable, compact and portable Raman spectrometers adaptable to different healthcare environments is necessary if SERS is to be adopted as a viable diagnostic system [36]. The significant improvement in component power, quality, and equipment performance, accompanied by a lower price and compact size, improves the feasibility of SERS for general and POC deployment [56]. Inasmuch as SERS is effective as a standalone technique, developing multimodal Raman spectrometers integrated with efficient sample handling techniques can enhance automation and protocol standardisation [134]. Beyond hardware improvements, advanced software and computational approaches can help overcome challenges associated with complex SERS data analysis and improve diagnostic accuracy. In particular, integrating data-driven and knowledge-based algorithms may enhance both the interpretability and reproducibility of SERS results [36,123].

Within the SERS research landscape, cutting-edge advances are being applied to resolve the identified challenges. For example, microfluidic integration represents an opportunity that simultaneously addresses matrix complexity, measurement reproducibility, and point-of-care applications [277]. Platforms such as the cardiac troponin I detection system reported by Campu et al. [95] and the acoustofluidic multimodal system for Alzheimer’s biomarker isolation developed by Hao et al [100] demonstrate that microfluidic sample handling can reduce protein corona formation, control analyte concentration at the SERS-active surface, and enable automated sequential detection without requiring skilled operator intervention. The extension of such platforms to include on-chip sample pretreatment (dilution, pH adjustment, filtration) would further reduce pre-analytical variability and bring SERS-based plasma diagnostics within reach of near-patient or point-of-care deployment. The fabrication costs of microfluidic SERS chips have decreased substantially with advances in soft lithography and injection moulding, and their scalability for clinical-volume production is no longer a prohibitive constraint. Also, microfluidic configurations with multiplex capability, such as spatial multiplex, barcode multiplex and Label-free multiplex, are significant and have the potential to simultaneously detect a panel of disease biomarkers [277]. Cutting-edge fibre-optic technology is being adapted for biosensing applications, where readily functionalised fibre surfaces can be integrated with SERS to provide highly selective light–matter interaction depending on various transduction mechanisms.

Multimodal detection strategies offer an empirical route to minimise the false-positive and false-negative rates that limit single-mode SERS platforms. As demonstrated in Section 5, combining SERS with complementary transduction mechanisms like electrochemical, colourimetric, photothermal, or acoustic ones enables orthogonal result validation within a single measurement workflow. It also reduces the impact of substrate-induced signal variability on diagnostic conclusions and can access biomarker information that is not spectrally accessible by SERS alone. The development of multimodal platforms is not restricted to highly sophisticated laboratory systems, as exemplified by the SERS–colourimetry lateral flow strip reported by Shende et al. [124] for simultaneous codeine and fentanyl detection in plasma without sample pretreatment. Simple dual-mode confirmation can be incorporated into formats compatible with decentralised testing.

AI-driven data analysis with clinical-grade validation presents an immediate opportunity to improve the translational credibility of existing SERS datasets. Many of the plasma studies reviewed in Section 5 contain spectral datasets of sufficient depth to support rigorous validation frameworks extended to real patient/sample cross-validation, large external cohort testing, and explainability mapping. Retrospective analyses of archived SERS datasets are capable of creating a comparative benchmark (currently unavailable) that can be used to determine viable clinical evaluation. Remarkably, the array of AI-based algorithms discussed in Section 6—with many models tailored to spectroscopic data analyses, for example, PSE-LR, RamanSpy, PyFasma, SSNet, and RADAR—is helping make SERS more quantitative.

The path to the development of a new diagnostic testing technology has several stages [56]. Stage 1 begins with a foundational scientific innovation, which typically originates from curiosity-driven exploratory research. Stage 2 utilises proof-of-concept studies to gather preliminary data that demonstrates the technology’s potential to outperform existing diagnostics or achieve lower limits of detection. Stage 3 focuses on establishing a market foothold by addressing initial manufacturing costs, streamlining experimental protocols, and validating performance on a small cohort of patients. Stage 4 involves the formal engineering design and physical construction of a functional prototype device or testing platform. Stage 5 executes large-scale clinical validation by employing biostatistical analysis to determine necessary sample cohorts and precisely quantify overall diagnostic accuracy. Stage 6 evaluates practical clinical utility by assessing how seamlessly the diagnostic technology integrates into existing laboratory workflows or point-of-care environments. And Stage 7 conducts real-world deployment trials designed to optimise the environmental stability, storage shelf-life, and robust performance of the assay. What is apparent from the progression through the stages is that most SERS-based research is at the development stage (Stage 2), where the LOD achieved for many SERS assays supports the viability of the technique. However, the pathway from a validated laboratory SERS assay to a clinically deployed diagnostic device has prerequisites that must be met, for example, performance validation and clinical performance data. Hence, the emphasis on overcoming the challenges that hinder SERS result reproducibility, which is key.

On a positive note, the different cohort studies point to unrelenting efforts being made towards the standardisation of SERS experimental protocols and measurement parameters [273,274,275], with appreciable progress achieved in the drive towards improving consistency in measurements and reliability in SERS assays. The recommendation is for unfettered access to complete technical information on spectrometer brands by their respective manufacturers; likewise, researchers providing complete research data, whether processed or raw, alongside extensive interlaboratory collaboration streamlined towards resolving key challenges in SERS should be re-emphasised [267]. Access to detailed information from the research community and equipment manufacturers is vital for the proper assessment of completed research, for extant challenges, and for the resolution of identified loopholes.

## 8. Conclusions

This review has presented a systematic account of SERS-based disease diagnostics in blood plasma organised around the three pillars that collectively determine the clinical viability of any SERS assay, namely the plasmonic Au and Ag NPs that generate signal enhancement, the biological matrix properties that govern what signals are accessible, and the computational strategies that extract diagnostic information from complex spectral data.

The evidence surveyed demonstrates that SERS is analytically competent across a broad disease landscape when assessed in controlled research settings. Label-free plasma diagnostics using colloidal AgNP and AuNP substrates have achieved diagnostic sensitivities and specificities that compare favourably with established screening tools: the 96.7% sensitivity and 92.0% specificity reported for cervical cancer discrimination using PCA-LDA [163], the 90.0% sensitivity for breast cancer versus the 70.0% benchmark of mammography [165], and the 89.8% accuracy for acute myeloid leukaemia subtype classification across 222 plasma samples [10] are representative of a broader pattern in which SERS performance, where rigorously validated, approaches or exceeds that of conventional first-line diagnostics. Labelled and immunoassay-formatted SERS platforms have extended this capability to quantitative single-analyte detection, achieving limits of detection at picogram mL^−1^ concentrations for cytokines [78], cancer antigens [168], and neurodegeneration biomarkers [18], with detection ranges that align with clinically actionable concentration windows.

What distinguishes plasma from other biological matrices examined in the SERS literature, and what this review has attempted to make explicit, is that the plasma matrix itself is simultaneously the source of diagnostic value and the primary source of analytical difficulty. The same protein abundance that makes plasma the most comprehensive biomarker repository in the body generates a protein corona that competes with target analytes for hotspot access. The strategies reviewed for managing these effects, for example, filtration, pH adjustment, electrophoresis-assisted fractionation, anisotropic substrate engineering, internal standard calibration, and multimodal detection, represent a fraction of the expansive methodology available to SERS assays towards addressing key limitations of a complex plasma matrix.

The integration of AI-based machine learning and deep learning with SERS data analysis has substantially extended the discriminative capacity of plasma SERS measurements in recent years. The progression from PCA-LDA applied to dozens of plasma samples toward automated ensemble classifiers and deep convolutional architectures validated in multi-centre cohorts of hundreds to thousands of participants reflects a genuine advance in methodological maturity. The demonstration that explainable AI methods can map classifier decisions back to specific Raman bands for the identification of molecular species is particularly significant for clinical translation because it bridges the gap between statistical performance and biochemical interpretability required by regulatory bodies.

Nevertheless, the overarching assessment from this review is that reproducibility remains unresolved at a systemic level. No SERS substrate type has demonstrated batch-to-batch performance consistency equivalent to the analytical standards applied to immunochemical platforms. No preprocessing or classification pipeline has been validated across independent laboratory platforms with sufficient rigour to support claims of cross-instrument generalisability, and no prospective multi-centre clinical trial of a SERS-based plasma diagnostic has been reported for any of the disease applications reviewed. The progress already achieved supports SERS as a clinically viable technique. Interlaboratory collaboration studies [267,273,274,275] have identified and in part quantified the sources of measurement variability. Microfluidic integration, advanced substrate fabrication, and AI-assisted analysis have each demonstrated their capacity to individually improve SERS diagnostic performance in plasma and other biological matrices. It is encouraging that the progress of SERS from academia to actual application has gone past the preliminary development stages, with some developed assays and devices already at the prototype and large-scale validation/testing stages. It is only a matter of time before all the concerted efforts by the SERS research community towards improving reproducibility, boosted by advances in technology, result in viable SERS assays and devices becoming one of the conventional clinical laboratory and POC tools.

## Figures and Tables

**Table 1:** Comparative overview of direct (label-free) and indirect (labelled) SERS detection strategies for plasma-based disease diagnostics.

Feature	Direct/Label-Free SERS	Indirect/Labelled SERS
**Signal origin**	Intrinsic Raman scattering of analyte molecules	Raman scattering of an exogenous reporter molecule on the NP surface
**Prior biomarker knowledge**	Not required; holistic profiling possible	Required; assay is designed around a specific target
**NP surface derivatisation**	Not required (bare NP) or bioreceptor only	Required; reporter molecule and targeting ligand
**Typical assay format**	Colloidal suspension or solid support, direct mixing	Sandwich immunoassay, aptasensor, or lateral flow
**Multiplexing capacity**	Limited by the spectral overlap of plasma constituents	High; multiple non-overlapping reporters detectable simultaneously
**Sensitivity**	High; limited by matrix interference and protein corona	Very high; reporter signal amplified and independent of matrix
**Specificity toward the target**	Moderate; dependent on chemometric discrimination	High; governed by biorecognition element selectivity
**Susceptibility to matrix effects**	High (protein corona, competitive adsorption)	Moderate (non-specific binding, corona on labelled NP)
**Sample preparation complexity**	Low to moderate (dilution, filtration, pH adjustment)	Moderate to high (nanotag synthesis, conjugation validation)
**Chemometric/AI requirement**	Essential for complex matrix discrimination	Moderate; calibration against the reporter band is sufficient for quantification
**LOD achievable in plasma**	pmol/L to nmol/L range (biomarker-dependent)	fg/mL to pg/mL range with immunoassay format
**Time to result**	Short (minutes to ~1 h)	Moderate (incubation and washing steps required)
**Suitable disease applications**	Unknown or non-specific biomarkers; exploratory profiling; cancer liquid biopsy	Quantification of known protein, nucleic acid, or metabolite biomarkers
**Primary limitation**	Protein corona; inter-sample spectral variability	Nanotag fabrication complexity; batch reproducibility of bioconjugation

**Table 2:** Comparison of physicochemical and analytical properties of the most common SERS-active nanoparticle systems used in plasma-based diagnostics.

Property	AuNP	AgNP	Au-Ag Hybrid (e.g., Au@Ag Core–Shell)
**LSPR range—spheres (nm)**	515–570	380–450	400–550 (tunable by shell thickness)
**LSPR range—anisotropic (nm)**	600–1300 (NIR-active)	450–900	450–1000
**Intrinsic SERS enhancement factor**	10^6^–10^8^	10^8^–10^10^	10^7^–10^10^
**Chemical stability in plasma**	High (resistant to oxidation)	Moderate (surface oxidation possible)	High (Au core provides stability)
**Surface functionalisation**	Excellent; thiol and amine chemistry is well-developed	Good; thiol chemistry; less robust than Au	Excellent; Au surface chemistry applicable
**Preferred excitation wavelength**	633–785 nm (red/NIR)	514–633 nm (green/red)	514–785 nm (tunable)
**Primary synthesis routes**	Citrate reduction; seed-mediated; electrochemical	Borohydride or hydroxylamine reduction; seed-mediated	Sequential shell growth on Au core
**Aggregation tendency in plasma**	Moderate; surface charge dependent	Higher; more susceptible to electrolyte-driven aggregation	Moderate (similar to Au)
**Preferred diagnostic application**	Labelled immunoassays; NIR tissue applications; label-free with >100 nm spheres	Label-free plasma profiling; high-sensitivity detection	Multiplex immunoassays; hybrid sensing platforms
**Key limitation**	Lower intrinsic EF than AgNP	Stability	More complex synthesis; increased cost

**Table 3:** Most common nanoparticle geometries used in SERS-based plasma diagnostics: structural characteristics, enhancement properties, and analytical relevance.

Geometry	Hotspot Generation Mechanism	EF Range	Key Synthesis Method	Primary Diagnostic Advantage	Principal Limitation
**Nanosphere**	Aggregation-dependent interparticle gaps	10^6^–10^8^ (aggregated)	Citrate or borohydride reduction	Simple synthesis; well-characterised; widely validated	Low single-particle EF; aggregation-dependent signal variability
**Nanostar**	Multiple branch tips (“lightning rod effect”)	10^9^–10^11^ (single particle)	Seed-mediated; surfactant-free reduction	High single-particle EF without aggregation;	Polydisperse; challenging synthesis reproducibility
**Nanorod**	Longitudinal tip enhancement; end-to-end junctions	10^7^–10^10^	CTAB-mediated seed growth	Tunable NIR LSPR; strong directional enhancement	Requires ligand exchange for bioconjugation
**Nanobipyramid**	Multiple sharp tips	10^9^–10^11^	Seed-mediated	Superior EF vs. nanorods; suitable for imaging and biomarker detection	Complex synthesis; limited commercial availability
**Nanoplate/Nanotriangle**	Sharp corners; edge hotspots	10^8^–10^10^	Chemical reduction; photochemical	Large surface area; high field enhancement; easy functionalisation	Difficult monodisperse synthesis; prone to shape transformation
**Nanowire**	End hotspots; inter-wire junctions	10^7^–10^9^	Electrochemical; template-directed	Large surface area; 3D substrate integration	Orientation-dependent signal; complex substrate integration

**Table 4:** Comparative overview of biological matrices other than blood plasma or serum in SERS-based diagnostics: collection characteristics, biomarker content, and matrix-specific analytical considerations.

Matrix	Collection Invasiveness	Primary Biomarker Classes	Matrix Complexity	Key SERS Advantage	Key SERS Challenge
**Tissue**	Invasive (biopsy)	Structural proteins; tumour markers; metabolites	Very high	Spatial in situ imaging; morphological correlation	Requires NP delivery to tissue; ex vivo limitations
**Hair/Nail**	Non-invasive	Metabolites; xenobiotics; trace elements	Low	Stable matrix; long historical window	Limited to forensic/toxicological applications
**Faeces**	Non-invasive	Microbial metabolites; host metabolites	Very high	Non-invasive; gastrointestinal biomarkers	Extreme compositional variability
**Sweat**	Non-invasive	Glucose; uric acid; creatinine; cortisol; electrolytes	Low	Non-invasive; wearable sensor compatible	Low analyte concentrations; contamination risk
**Tears**	Minimally invasive	Lysozyme; hundreds of proteins; lipids; metabolites	Moderate	Non-invasive; ocular and systemic biomarkers	Very small collection volumes
**Saliva/Sputum**	Non-invasive	Proteins; nucleic acids; pathogens; metabolites	Moderate to high	Non-invasive; oral and respiratory access	Contamination; diurnal variation
**Urine**	Non-invasive	Creatinine; uric acid; glucose; proteins; nucleic acids	Low to moderate	Large volume; low protein; non-invasive	Dilute analytes; variable concentration
**CSF**	Highly invasive (lumbar puncture)	Neurodegenerative markers; pathogens; metabolites	Low	Low complexity; CNS-specific biomarkers	Invasive collection; limited volume
**Whole blood**	Minimally invasive	All classes (cellular + plasma)	Very high	Complete biomarker representation	Cellular components complicate NP interaction

**Table 5:** Summary of some direct/label-free and indirect/labelled SERS assays for different pathologic conditions in blood plasma.

Substrate	Target Analyte	Pathology	Chemometrics	Total Sample	Ref.
AuNP Sp colloid	Antiretroviral drug: Emtricitabine (FTC)	HIV ART compliance	Q_i_ CDF, PCA	-	[20]
AgNP Sp Colloid	Plasma	Nasopharyngeal cancer	PCA LDA	76	[173]
Colloidal AgNP Sp	Plasma	Gastric cancer	PCA LDA	65	[174]
AgNP Sp colloid	Plasma	Cervical cancer	PCA LDA	110	[163]
AgNP Sp colloid	Plasma	Colorectal cancer	PLS LDA	69	[87]
AgNP Stars	Plasma	Stroke	PCA Light GBM	NA	[17]
AuBP@Ab	Cardiac troponin I (cTnI)	Acute myocardial infarction (AMI)	PV (NPV, PPV)	80	[95]
AuNP Sp@5-CB	Glucose	Diabetes	PCA LDA	30	[167]
BC@4-MP@Ag NP	Plasma	Colorectal cancer	PCA, ML (DT, KNN, RF, SVM)	40	[121]
3D-AgNP@Polymer	Plasma	Kidney and bladder cancers	PCA LDA	66	[49]
AuNW, SAM, 6E10 Ab.	Aβ(1-42) & metabolites	Alzheimer’s	DL (ffNN), AI (IG)	40	[18]
AgNP Sp	Plasma	Acute myeloid leukaemia	CRT, ANOVA	222	[10]
Au bipyramid@PLFS	S-100β	TBI (traumatic brain injury)	Linear regression analysis	NA	[32]
Au_ZnO@Ag@anti-Aβ42/anti-tau	Aβ peptides, tau proteins	Alzheimer’s disease	LDA	17	[100]

**Table 6:** Contemporary cohort review summary.

Computational Category	Papers	Primary Model Implementations	Common Deep Learning Features	Evaluation Metrics	Observed Accuracy and Performance Range
Classical Machine Learning & Statistical Discriminants	[49,127,210,211,216,217,219,220,225,227,228,253]	Support Vector Machines (SVMs) Linear Discriminant Analysis (LDA) Partial Least Squares (PLS-DA) Random Forests (RF) Decision Trees (DT)	None (Relies entirely on pre-extracted numerical features)	Sensitivity/Recall Specificity Overall Accuracy ROC/AUC Indices Confusion Matrices	84% to 100% (Typically achieving >90% for well-separated clinical conditions)
Deep Learning	[236,241,259,261,262,263]	Custom 1D-CNNs Restructured 2D-CNNs Multilayer Perceptrons (MLP/ANN)	1D/2D Convolutional Blocks Batch Normalisation (BN) Max-Pooling Layers Dropout Regularisation SoftMax Output Layer	F1-Score Precision/Recall Probability (Porotein) Classification Success Rate (CSR) Limit of Detection (LOD)	95% to 98.5% (Highly stable and effective without requiring manual feature selection)
Advanced Pre-trained & Residual Backbones	[177,239]	Deep Residual Networks (ResNet/ResNet18) Multiscale Fusion CNN (MCNN) Fine-tuned Visual Backbones (AlexNet, VGGNet, GoogLeNet, SqueezeNet)	Residual Skip-Connections Multi-scale Variable Kernel Size Deep Hidden Layers (100–2500+ units) RAdam Optimisers	Staging-Specific Accuracy Diagnostic Sensitivity Area Under the ROC Curve (AUC)	86% to 98% (Lower end represents complex sub-disease staging; higher end represents binary diagnostic splits)
Data Augmentation, Resampling & AutoML Pipelines	[231,232,247]	Synthetic Balancing (SMOTE, BorderlineSMOTE, ADASYN) Hybrid Cleaners (SMOTEENN, SMOTETomek) Outlier Filters (PCA-DBSCAN) Automated Ensemble Stacking (AutoGluon)	Deep Neural Networks (DNN) Hyperparameter-tuned Ensembles Automated Multi-layer Stacking	Balanced Accuracy Concentration Prediction Error Pearson Coefficient (R2) Cross-Cohort Parity	93% to 100% (Optimised explicitly to handle highly unbalanced or highly asymmetrical clinical datasets)
Interpretability & Explainable AI (XAI) Frameworks	[18,232,247]	Feature Attribution Modalities Mathematical Peak Mapping	Integrated Gradients (IG) Class Activation Mapping (CAM/Grad-CAM) Game-Theoretic SHAP Values	Spectral Peak Localisation Contribution Heatmaps Validation against clinical baselines	94.7% to 100% (Provides transparency by tracing decision weights directly back to biochemical peaks)

**Table 7:** Structured overview of data paradigms in recent SERS-based AI studies.

Category	Subtype	Description	Representative Studies
Biological matrix	Liquid biopsy matrices	Spectra derived from serum, plasma, and urine used as primary diagnostic media	[49,212,217]
	Subcellular/vesicle-based systems	Isolation of exosomes or circulating vesicles to reduce biochemical background noise	[218,232,240]
	Controlled/spiked systems	Synthetic or controlled environments (animal serum or spiked drug solutions) for calibration and mechanistic modelling	[49,222,253]
Cohort scale	Exploratory clinical datasets	Small-scale patient cohorts used for proof-of-concept modelling	[18,213,216]
	Expanded spectral representations (“patient-to-spectrum inflation”)	Multiple spectral acquisitions per patient used to augment dataset size for deep learning training	[44]
	Large-scale clinical cohorts	Multi-centre or high-sample datasets enabling population-level validation	[212,232,247]
Validation strategy	Static holdout splits	Fixed train/test partitions (e.g., 70:30, 80:20) used for baseline evaluation	[31,39,240]
	k-fold cross-validation	Iterative resampling (typically 5- or 10-fold) for robustness under limited sample sizes	[18,216]
	External/allopatric validation	Independent geographically separated cohorts used for true generalisability testing	[212,232]

## Data Availability

Not applicable.
